# Potential sex-dependent effects of weather on apparent survival of a high-elevation specialist

**DOI:** 10.1038/s41598-020-65017-w

**Published:** 2020-05-20

**Authors:** Eliseo Strinella, Davide Scridel, Mattia Brambilla, Christian Schano, Fränzi Korner-Nievergelt

**Affiliations:** 1 Reparto Carabinieri Biodiversità L’Aquila, L’Aquila, Italy; 2 Museo delle Scienze di Trento (MUSE), Sezione Zoologia dei Vertebrati, Corso del Lavoro e della Scienza 3, 38122 Trento, Italy; 3 Ente Parco Naturale Paneveggio Pale di San Martino, loc. Castelpietra, 2-Tonadico, Trento, Italy; 4 Fondazione Lombardia per l’Ambiente, Largo 10 luglio 1976 1, I-20822 Seveso, MB Italy; 5 Swiss Ornithological Institute, Seerose 1, CH 6204 Sempach, Switzerland; 6 University of Zurich, Department of Evolutionary Biology and Environmental Studies, Winterthurerstrasse 190, CH 8057 Zurich, Switzerland

**Keywords:** Climate-change ecology, Population dynamics

## Abstract

Mountain ecosystems are inhabited by highly specialised and endemic species which are particularly susceptible to climatic changes. However, the mechanisms by which climate change affects species population dynamics are still largely unknown, particularly for mountain birds. We investigated how weather variables correlate with survival or movement of the white-winged snowfinch *Montifringilla nivalis*, a specialist of high-elevation habitat. We analysed a 15-year (2003–2017) mark-recapture data set of 671 individuals from the Apennines (Italy), using mark-recapture models. Mark-recapture data allow estimating, forgiven time intervals, the probability that individuals stay in the study area and survive, the so called apparent survival. We estimated annual apparent survival to be around 0.44–0.54 for males and around 0.51–0.64 for females. Variance among years was high (range: 0.2–0.8), particularly for females. Apparent survival was lower in winter compared to summer. Female annual apparent survival was negatively correlated with warm and dry summers, whereas in males these weather variables only weakly correlated with apparent survival. Remarkably, the average apparent survival measured in this study was lower than expected. We suggest that the low apparent survival may be due to recent changes in the environment caused by global warming. Possible, non-exclusive mechanisms that potentially also could explain sexual differential apparent survival act via differential breeding dispersal, hyperthermia, weather-dependent food availability, and weather-dependent trade-off between reproduction and self-maintenance. These results improve our current understanding of the mechanisms driving population dynamics in high-elevation specialist birds, which are particularly at risk due to climate change.

## Introduction

Mountain ecosystems are recognised as global biodiversity hotspots, hosting highly specialized and endemic species^1–3^ which are threatened by human-induced causes including climate change^4–9^. Mountain regions are particularly susceptible to climatic alterations and are experiencing a faster rate of warming compared to the global average. Indeed, the European Alps have warmed about 2 °C in the past 100 years, with the largest increase occurring in the last three decades^4–6^. In parallel to changes in temperature, the frequency of extreme weather events is also increasing^10^, potentially enforcing detrimental effects of climate warming on organisms^11^.

Extreme environments, such as the alpine and nival belts of mountains, are often inhabited by highly specialized species that are adapted to local conditions^12^. Conditions at high elevations are characterised by low average temperature, strong winds, intensive sun radiation, low oxygen pressure, and a high temporal and spatial variation in temperature. Extremely warm temperatures (>25 °C in the European Alps) can be followed by cold temperatures and even snow storms within minutes. Species inhabiting these variable environments must show a high physiological and behavioural flexibility to cope with sudden abiotic changes within short periods of time, while they also need to be able to persevere with long-lasting inclement weather periods. Organisms being specialised to extreme environments may be vulnerable to changes in their habitats and climate for the following reasons. They may already live at the edge of their physiological niche, and even small shifts in one environmental or climatic factor may render an area unsuitable^13^. Their ecological niche may be narrow. Therefore, they may not be flexible enough to adapt their behaviour, ecology or life-history traits rapidly enough to cope with long-term and directed changes in the environment and climate^12,14,15^. At last, many alpine species have a limited distributional range: the loss of a few populations increases extinction risk of the species and consequently represents a threat to global biodiversity^16^.

In birds, the adaptations for living in alpine zones may be as manifold as there are species^17^, or even populations. Nevertheless, meta-analyses showed that populations at higher elevations have lower fecundity (number of breeding attempts and clutch size) but slightly heavier nestlings and higher juvenile survival compared to their conspecifics at low elevations (e.g.^18–20^). With regard to adult survival, we would expect that alpine species compensate the risk of unpredictable conditions during the reproductive season with a longer life span^19,21^. A long life span is characteristic for some alpine bird species (e.g. white-tailed ptarmigan *Lagopus leucurus* in the alpine zone of the Rocky Mountains compared to populations in the sub-alpine zone and Arctic^22^; an alpine subspecies of horned lark *Eremophila alpestris* compared to a lowland subspecies^23^). However, a long life span does not seem to be a universal characteristic for species living at high elevations^18,20,24,25^ and various calls have been made to improve basic knowledge on demographic parameters for the mountain bird community^25^. Improving the knowledge of demographic parameters, such as survival and reproduction, of a variety of different mountain bird species would be a crucial step for understanding how life-history traits of mountain birds are shaped by their extreme environment, and consequently understand the needs and vulnerability of their populations.

We studied apparent survival of a high-elevation bird species, the European subspecies of the white-winged snowfinch *Montifringilla nivalis nivalis* (hereafter snowfinch). It breeds in southern European mountains, exclusively above the treeline. In the Alps, the species has lost parts of its former distribution and population density decreased during the last decades^26–29^. There is evidence that global warming may be an important cause of this population decline: a comparison across species showed a correlation between thermal niche and changes in distribution ranges in Italy. The distribution of cold-adapted species, including the snowfinch, generally shrunk during the last 30 years, whereas species of warm habitats expanded their distribution^30^. Further, both distribution models^31,32^ and fine-scaled habitat selection studies^33,34^ suggested that the snowfinch is highly dependent on climate sensitive habitats (i.e. snow patches and short alpine grassland) and therefore it is potentially threatened by global warming.

The specific aims of this study are threefold. First, we estimate annual apparent survival for adult males, adult females and juveniles in order to fill a knowledge gap in the life-history of this high-elevation specialist in a southern part of its European distribution. Second, we assess the role of summer and winter temperatures as well as precipitation on males’ and females’ annual apparent survival. Third, we describe how apparent survival changes over the annual cycle in order to identify periods with increased mortality, i.e. key information to better understand the factors driving annual apparent survival. The findings of this study will improve the understanding of the mechanisms underlying demographic trends and life history traits for a poorly studied group of species adapted to extreme, dynamic and globally changing environments.

## Results

### Annual recapture probability and apparent survival

We analysed the data using a fully Bayesian approach. Our conclusions are based on the posterior distributions of the model parameters. In order to obtain posterior distributions by probability theory we had to make assumptions about the natural process that generated our data. We explored how different assumptions affected the results by using seven different models (Table 1) fitted to two different data subsets. From the posterior distributions of the model parameters we reported the median as a point estimate and the 2.5 and 97.5% quantiles as lower and upper limits of the compatibility interval^35^, which we abbreviated with CI. We tried to avoid drawing dichotomous conclusions, but discussed effect sizes while acknowledging various sources of uncertainty^36^.

**Table 1:** List of models used. In the brackets after $\varPhi$ the model for apparent survival probability is specified and in the brackets after *p* the model for recapture probability.

Name	Model for full data set	Model for reduced data set
1	$\Phi (sex\ast age)p(sex\ast year)$	$\Phi (sex)p(sex\ast year)$
2a	$\Phi (sex\ast age+sex|year)p(sex\ast year)$	$\Phi (sex+sex|year)p(sex\ast year)$
2b	$\Phi (sex\ast age\ast first+sex|year)p(sex\ast year)$	$\Phi (sex\ast first+sex|year)p(sex\ast year)$
3a	$\Phi (sex\ast age\ast ({T}_{su}+{T}_{wi}))p(sex\ast year)$	$\Phi (sex\ast ({T}_{su}+{T}_{wi}))p(sex\ast year)$
3b	$\Phi (sex\ast age\ast first\ast ({T}_{su}+{T}_{wi}))p(sex\ast year)$	$\Phi (sex\ast first\ast ({T}_{su}+{T}_{wi}))p(sex\ast year)$
23b	$\Phi (sex\ast age\ast ({T}_{su}+{T}_{wi})+first+sex|year)p(sex\ast year)$	$\Phi (sex\ast ({T}_{su}+{T}_{wi})+first+sex|year)p(sex\ast year)$
4	$\Phi (sex\ast age\ast ({T}_{su}+{T}_{wi}+P{r}_{su}+P{r}_{wi}))p(sex\ast year)$	$\Phi (sex\ast ({T}_{su}+{T}_{wi}+P{r}_{su}+P{r}_{wi}))p(sex\ast year)$
5	$\Phi (sex\ast age\ast season)p(sex\ast year\ast season)$	$\Phi (sex\ast season)p(sex\ast season)$

A “+” indicates an additive relationship, a “*” indicates a multiplicative (interaction) relationship. The “|”-sign (“grouped by”) indicates a random factor. The explanatory variables sex, age, year, season and first (indicator of first occasion) are categorical variables. Temperature (T) in summer (su) and winter (wi) and precipitation (Pr) are continuous numeric predictors.

We fitted seven different models once to the full data set, and once to a reduced data set with only individuals of which the sex was known and including only recaptures after the capture at which sex was first identified (see Methods). All models included separate apparent survival for age and sex classes, but they differed in the temporal structure for apparent survival. The simplest model (1) assumed constant annual apparent survival over the years. The most complex one (23b) included a specific apparent survival for the year after the first capture, random year effects for each sex separately, and linear effects of summer and winter temperature. We compared the performance of the models by predictive model checking^37,38^. Thereby, we specifically checked for transients (i.e. proportion of individuals captured only once^39^). Furthermore, we compared the number of individuals captured at least three times between the model prediction and the observed data. A lack of fit in the number of individuals captured at least three times would indicate either an under- or overestimation of apparent survival, or heterogeneity of capture probability (e.g. specific trap responses of some individuals^40^).

All models fitted to the reduced data set adequately predicted the number of individuals captured exactly once and the number of individuals captured at least three times (Table S1). The three models that accounted for transients (2b, 3b, and 23b) performed best. For the full data set, generally the models adequately predicted the observations for the individuals with known sex, except when including four environmental variables as predictors for apparent survival (summer and winter temperature and precipitation, model 4). However, for the individuals with unknown sex, only models accounting for transients (models 2b, 3b, and 23b) did reasonably well, though not perfect. These models predicted between 304 and 381 individuals that were captured only once, whereas the data contained 389 individuals captured once (Table S1). When including different effects of the environmental variables on apparent survival in the year after the first capture and later (interaction first capture x environmental variables), at least one of the estimated coefficients was highly uncertain (95% CI included the range between −1 and +1, which means that both strong negative as well as strong positive correlations were compatible with the data).

Parameter estimates were consistent among all models. If not otherwise stated, we presented the results from the model including summer and winter temperature as predictors for apparent survival as well as allowing for a sex specific among-year variance and accounting for transients (model 23b). We presented the results for both data sets. Results for all models fitted to both data sets are reported in Table S2.

Average recapture probability was similar between males and females, but varied strongly among years. Recapture probabilities ranged between 0.1 and 0.8 both in the full and reduced data set (average: 0.4). Estimated recapture probabilities for each year and sex were consistent between the models and the data sets (Pearson’s correlations among estimated recapture probabilities of different models were between 0.78 and 0.97).

**Figure 1:**
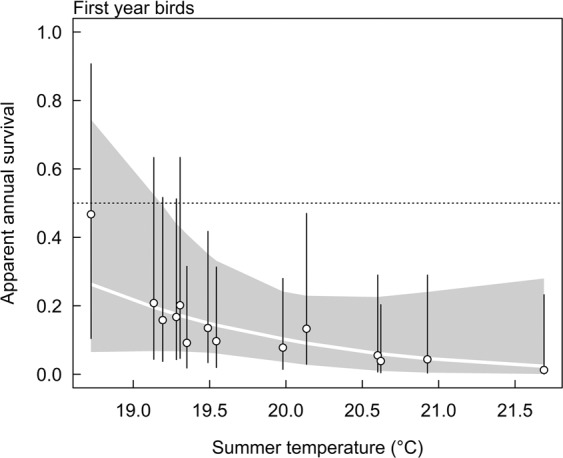
Annual apparent survival estimates for first year birds in relation to summer temperature. Circles are medians of posterior distributions obtained by model 2b, vertical bars connect the 2.5% and 97.5% quantiles of the posterior distributions (95% compatibility intervals). The regression line is based on model 3b. Grey shaded area is the 95% compatibility interval of the regression line. For juveniles, we cannot distinguish between first year after first capture and later years, because later they are adults. Horizontal dotted line is the mean of the prior distribution.

Apparent annual survival estimates were between 0.09 and 0.16 for nestlings and first year birds (Table 2). First year apparent survival may be negatively correlated with summer temperature (estimate: −0.76, CI: −2.22, 0.39, Fig. 1). Correlation with winter temperature was unclear (−0.16, CI: −1.24, 1.02).

**Table 2:** Annual apparent survival estimates for individuals ringed as nestlings, for first year birds (juveniles), adult males and adult females as estimated by different models fitted to the full and reduced data.

Model and data	Nestlings	Juveniles	$\varPhi 1$ Females	$\varPhi 1$ Males	$\varPhi 2+$ Females	$\varPhi 2+$ Males
2b full	0.13 (0.05,0.29)	0.13 (0.06,0.26)	0.35 (0.16,0.69)	0.26 (0.16,0.42)	0.59 (0.34,0.86)	0.50 (0.36,0.68)
3b full	0.12 (0.04,0.34)	0.09 (0.04,0.19)	0.33 (0.14,0.86)	0.26 (0.16,0.40)	0.54 (0.34,0.86)	0.54 (0.40,0.71)
23b full	0.12 (0.04,0.34)	0.11 (0.04,0.26)	0.38 (0.17,0.77)	0.28 (0.16,0.55)	0.64 (0.36,0.91)	0.52 (0.36,0.75)
2b red	—	—	0.35 (0.16,0.64)	0.28 (0.17,0.47)	0.53 (0.31,0.81)	0.47 (0.31,0.68)
3b red	—	—	0.33 (0.16,0.77)	0.26 (0.17,0.39)	0.51 (0.30,0.83)	0.44 (0.29,0.62)
23b red	—	—	0.34 (0.14,0.69)	0.29 (0.17,0.52)	0.57 (0.32,0.88)	0.49 (0.31,0.72)

Model 2b includes for each age, sex and year a separate annual apparent survival. The average over all years is given. Models 3b and 23b linearly relate annual apparent survival to summer and winter temperature. The estimated apparent survival given for these models are calculated assuming average summer and average winter temperature. Compatibility intervals are given in parentheses.

For adults, annual apparent survival was between 0.26 and 0.28 for males and between 0.33 and 0.38 for females during the first year after the first capture (Table 2). During later years, apparent survival was between 0.44 and 0.54 for males and between 0.51 and 0.64 for females. Apparent survival was slightly but consistently higher for females compared to males. Females showed a larger among-year variance in apparent annual survival (standard deviation among years in full data: 1.41 (CI: 0.27, 3.16) for females, and 0.43 (CI: 0.02, 1.34) for males; in reduced data: 0.87 (CI: 0.05, 2.92) for females, and 0.48 (CI: 0.02, 1.68) for males, taken from the model not accounting for temperature, model 2b).

**Figure 2:**
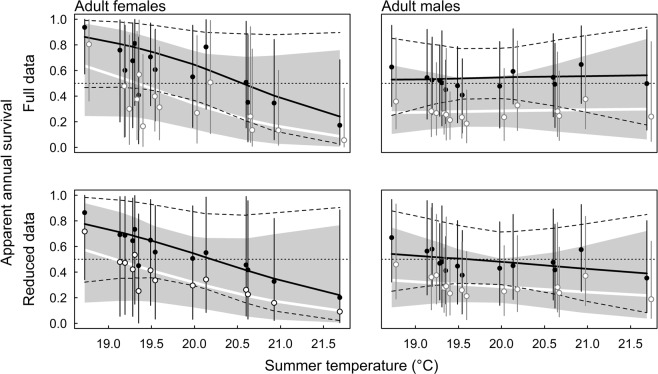
Annual apparent survival estimates for adult females and males against mean summer (months June to September) temperature based on the full data set (upper panels, all data and accounting for individuals with unknown sex within the model) and the reduced data set (lower panels, only including data of individuals with known sex and only including capture and recapture occasions after their sex has been identified). Open circles and white regression line are apparent survival estimates in the year after first capture, filled circles and solid regression line relate to later years. Shaded area and broken lines indicate 95% compatibility intervals of the regression lines, vertical bars of the annual apparent survival estimates. Dotted horizontal line corresponds to the mean of the prior.

When including both precipitation and temperature as predictors for apparent survival (model 4), posterior distributions of the model coefficients became broad. The clearest correlations were a negative one between female apparent survival and summer temperature (−0.85, CI: −2.09, 0.22) in the full data set, and a positive correlation (1.24, CI: −0.29, 3.22) between female apparent survival and summer precipitation in the reduced data set. In both data sets, summer temperature was negatively and summer precipitation positively correlated with female apparent survival (Table S2). However, CIs were so broad that we cannot clearly conclude that both variables independent of the other correlate strongly with female apparent survival. Further, summer temperature and precipitation were negatively correlated (Pearson’s correlation coefficient −0.39). Therefore, we present the correlation between summer temperature and apparent survival from models that only include temperature as predictor for apparent survival keeping in mind that warm temperatures also mean dry summers (Fig. 2). In both data sets, we found clear negative correlations between summer temperature and apparent survival of females during their first year after first capture (full data: −1.12, CI: −2.53, −0.08; reduced data: −1.07, CI: −3.05, −0.16), whereas for males, this correlation does not seem to be so strong (full data: 0.03, CI: −0.55, 0.67; reduced data: −0.15, CI: −0.70, 0.42). For later years, the CI of the correlation between apparent survival and summer temperature included both strong positive and strong negative values. When assuming that the effect of temperature does not differ between the first and later years after first capture, the correlation between temperature and female apparent survival was negative (model 3a full data: −0.78, CI: −1.75, 0.03, reduced data: −0.70, CI: −1.61, 0.07; when accounting for additional among year variance (model 23b) full data: −0.85, CI: −2.18, 0.42; reduced data: −0.72, CI: −1.98, 0.62). For males, the correlation between summer temperature and apparent survival was probably only weak (model 3a full data: 0.18, CI: −0.47, 0.92, full data: −0.17, CI: −0.75, 0.42; accounting for additional among year variance (model 23b) full data: 0.05, CI: −0.79, 0.98; reduced data: −0.18, CI: −1.11, 0.68). The posterior probability of the hypothesis that female apparent survival shows a stronger negative correlation with summer temperature than males is 0.90 based on the full data set and 0.79 in the reduced data set (model 23b). When only looking at apparent survival during the first year after the first capture, females clearly show a stronger negative correlation with summer temperature (posterior probability 0.97 in the full data and 0.95 in the reduced data).

Correlations of winter temperature with apparent survival were generally less clear, but a positive correlation for females was evident when assuming that winter temperature affects apparent survival during the first year after first capture similarly as during later years and not allowing for additional among year variance (model 3 full data: 0.99, CI: 0.02, 2.66, reduced data: 0.44, CI: −0.26, 1.36).

### Seasonal recapture probability and apparent survival

Four-month recapture probability was highest during the breeding season (full data 0.18, CI: 0.09, 0.46 similar for males and females, reduced data 0.23, CI: 0.13, 0.41 for males and 0.2,2 CI: 0.11, 0.40 for females). Between August and March, four-month recapture probability varied between 0.03 and 0.12.

**Figure 3:**
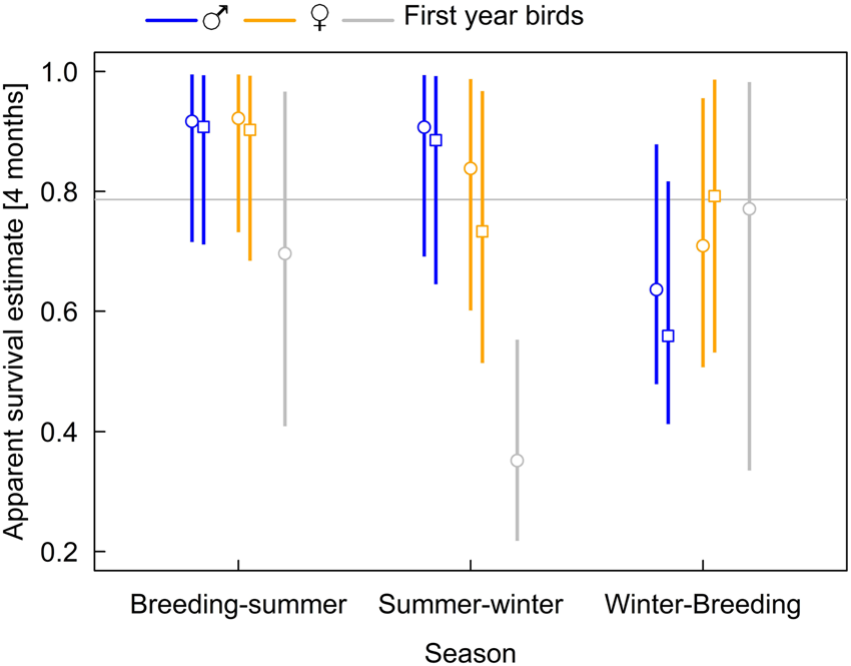
Seasonal (4-months) apparent survival estimates of adult males (blue), females (orange), and first year birds (grey). Circles are based on the full data set, squares are based on the reduced data set. Given are medians of the posterior distributions, vertical bars are 95% compatibility intervals. Grey horizontal line indicates the median of the prior distribution Beta(3.6, 1.2). Deviations of the estimates from this median indicate information in the data. Winter: December–March; breeding: April–July; summer: August–November.

Apparent seasonal survival estimates were similar between the full and reduced data set. For adult males apparent survival was high from breeding to winter and clearly lower from winter to breeding (Fig. 3). For females, already autumn survival was lower than during summer and it stayed low for the winter (Fig. 3).

Of first year birds, a proportion of 0.70 (CI: 0.41, 0.97) survived and stayed in the study area until summer and of those 0.35 (CI: 0.22, 0.55) survived and stayed until their first winter. Thus, a proportion of 0.24 (CI: 0.14, 0.39) of first year birds ringed during the breeding time were still alive in the study area the following winter. The estimate of apparent survival of juveniles from winter to the next breeding season showed large uncertainty. However, given that apparent annual survival of first year birds was around 0.10–0.15, we can expect that a proportion of around 0.5 of those individuals alive and present in winter will survive and stay in the study area until the next breeding season.

## Discussion

The strong among-year variance in annual recapture probabilities may reflect the strong among-year variance in snowfinch breeding and spatial behaviour driven by strong variance in weather and food conditions typical of high elevation environment^41^. The capture effort, measured as the number of field days, was fairly constant across years. However, capture effort was much higher in summer compared to winter. Additionally, during the breeding season, snowfinch spatial behaviour is easier to predict because they are involved in reproduction, explaining the higher capture probability during the breeding season compared to the rest of the year.

The average apparent annual adult survival estimated in this study for snowfinches based on mark-recapture data from 671 individuals in the Central Apennines was around 0.50 for males and between 0.51 and 0.64 for females. Such apparent survival estimates seems to be lower compared to earlier similar measures for snowfinches in the Eastern Alps. Lindner (2002)^42^ reported that, out of 24 breeding birds, 14 (a proportion of 0.58) returned in the next breeding season. From 482 birds ringed in the Austrian Alps during the years 1973–1994 by A. Aichhorn, 52 were recaptured later^43^. The mean age of these recaptured birds was 4.4 years (oldest bird was 14 years), and 12 out of 52 birds were at least 6 years old when they were recaptured. In our data, none out of 138 recaptured birds was older than 6 years. Thus, the annual adult apparent survival measured in this study is very likely substantially lower than it has been in the Austrian Alps 30 years earlier. Also, a comparison with the phylogenetically related, but 30% smaller, house sparrow *Passer domesticus* suggests that we could expect a higher apparent survival than the one we measured. Based on a mark-recapture data set from Norway, Holand *et al*.^44^ estimated an apparent annual survival between 0.6 and 0.7 for the house sparrow. According to allometric relationships we would expect that the snowfinch has a higher survival compared to the house sparrow^45,46^.

Our estimate of annual apparent survival may be lower than expected or measured elsewhere because of methodological or ecological reasons. In our analyses, we might not have accounted for all capture heterogeneity. Capture heterogeneity is present when groups of individuals have a different probability of being captured. Not accounting for capture heterogeneity in a mark-recapture model can lead to an underestimation of survival^47^, e.g. if weak individuals are captured with a higher probability. More interestingly, snowfinches in our study area may show a lower apparent survival than those in the Alps because, in the Central Apennines, dispersal rates are higher or the average life span is shorter. Reasons for this difference could be unfavourable environmental conditions, or local adaptations of life-history characteristics.

Our models accounted for differences in capture probability and apparent survival between age and sex classes, first year birds, adult males and adult females, as well as among years and seasons. We did not account for potential differences between different age classes among adults, because exact age was only known for a few individuals ringed as nestlings, nor did we relate capture probability to body condition. However, bias produced by mist-nets capturing weak birds with a higher probability than strong birds must have occurred also 30 years earlier in Austria. Therefore, we do not think that the difference between our estimate of apparent annual adult survival and those for Austrian snowfinches 30 years earlier can be explained by unaccounted heterogeneity in capture probability alone.

Low apparent survival could have resulted from local adaptations of the life-history traits in snowfinch populations of the Apennines (e.g.^19^). Because of the more southern latitudes of the Apennines compared to the Alps, the summer seasons always were warmer and longer compared to the Alps, probably providing more time and better conditions for the broods. Average clutch size may be slightly higher in the Apennines (mean 4.4, range 3–5, n = 48^48^); compared to the Alps (mean 3.9 eggs, range 2–6, n = 33, own unpublished data). Additionally, the proportion of second broods may be higher when the season is longer but no data on the proportions of second broods is available. It may be that snowfinch populations in the Apennines invest more energy in reproduction than in survival as an adaptation to local conditions. Alternatively, snowfinches in the Apennines may naturally disperse more often after breeding compared to snowfinches in the Alps. Indeed, the lower apparent survival of adults during the first year after first capture compared to later indicates that parts of the individuals captured at the study sites are not staying in the study area. However, even after having accounted for such transient individuals in our models, apparent survival estimates were still unexpectedly low. Maybe snowfinches in the Central Apennines regularly disperse also after having stayed for some years, e.g. after having experienced low breeding success^49,50^. Further, breeding dispersal in birds is generally higher in females compared to males^51–53^, leading to a lower apparent survival in females compared to males. We cannot see lower apparent survival in females compared to males in our data (Table 2). Therefore, either the snowfinches in the Central Apennines show breeding dispersal patterns not typical for birds, or breeding dispersal may be low and the apparent survival estimates presented here for the second year and later after first capture may be close to true survival. To what extent snowfinches in the Central Apennines perform breeding dispersal clearly needs further investigations.

Obviously, local conditions in the Apennines have changed dramatically during the last decades: mean annual temperature increased by 2 °C within the last 60 years and snow precipitation decreased by 50% during the last decade in our study area^54^. Thermophilic and nutrient-demanding plant species became more abundant, whereas cold-tolerant plant species declined in the Apennines during the last 42 years^54–56^. Consequently, quantity and quality of seed availability (main snowfinch food, exclusively in winter) and accessibility of ground living insects (important nestling food) have presumably changed during the last decades. Such changes in food availability have the potential to negatively affect survival and/or positively affect breeding dispersal behaviour due to low breeding success. Therefore, our results may complement the many studies that showed population declines of mountain birds due to habitat loss induced by climate change^9,57,58^. The low adult apparent survival found in this study may indicate that, for the snowfinch, climate-induced population declines may act, beside other mechanisms, via reduced survival of adults or increased emigration.

Its strong among-year variance suggests that female apparent survival is dependent on weather. Indeed, snowfinch female apparent survival was much lower in years with warm and/or dry summers, but less so in males. We further showed that adult apparent survival is lower during winter compared to summer. Therefore, we would expect that weather conditions during winter are more important in defining annual apparent survival compared to summer weather conditions. However, at least for females, summer conditions showed a stronger correlation with annual apparent survival than winter weather conditions.

Compared to males, snowfinche females are slightly smaller (1% in body mass, 5% in wing length^59^). The eggs are exclusively incubated by females^60^. Both parents feed the young but females presumably more intensively than males, e.g., as observed in the house sparrow^61^. Overall, in snowfinches reproductive investment seems to be substantially higher for females compared to males. Warm and dry summers may have direct or indirect effects on apparent survival potentially differently in male and female snowfinches via 1) hyperthermia, 2) food availability and accessibility in winter, 3) trade-off between reproduction and self-maintenance.

First, hot and dry weather conditions can cause physiological problems due to dehydration or hyperthermia. Birds adapted to living in cold climate seem to be particularly at risk to hyperthermia. For example, in ptarmigans (*Lagopus muta* and *L. leucurus*), body temperature and evaporative water loss increased at temperatures above 30 °C^62,63^. In direct sunlight ptarmigans actively seek shelter from sun even at much lower temperatures (i.e., above 21 °C^64^). High temperatures can cause direct mortality through hyperthermia and dehydration or reduce the time for foraging and maintenance because of the need for seeking shelter^65^ and therefore indirectly increase mortality. Heat stress may affect females more strongly than males, because of their different roles in the rearing of the brood.

Second, the main food of adult snowfinches is represented by wildflower seeds, particularly in winter^66,67^. During warm and dry summers the seed production of wild flowers can be lower compared to cool and wet summers (e.g. *Campanula thyrsoides*^68^). Therefore, summer conditions may affect food availability in the following winter and thus may affect survival or dispersal in winter. During the winter, snowfinches usually forage in flocks where individuals compete when food is scarce^69^. In case of competition, males may dominate over females, and therefore food shortage may affect females more severely than males^70^. For the Alpine cough *Pyrrhocorax graculus* that inhabits similar habitat to the snowfinch, Chiffard *et al*.^71^ recently also hypothesised that food shortage could lead to lower survival in females compared to males due to competition. Further, males are slightly larger than females. A larger body size may be of advantage for persevering with food shortage, or when access to food is more difficult because of the snow layer preventing or impeding access to seeds.

Third, warm summers may increase reproductive effort either by allowing second broods or by an increase in effort needed to raise a brood. During years with medium to early snow melt, an unknown proportion of breeding pairs lay a second clutch^60,72,73^, but when snow melt is late, snowfinches can even skip breeding^41^. Therefore, in warm and dry summers, we expect a higher proportion of breeding pairs raising two broods. On the other hand, nestling food availability may be reduced during warm and dry summers because snow patches vanish quickly^33^. Along the edges of melting snow patches, snowfinches forage for Tipulidae larvae that constitute the most important food for their nestlings^41^. Broods raised in close proximity to melting snow patches have higher breeding success compared to broods without snow patches in close vicinity^41^. A lack of melting snow patches during the rearing period (mid May to mid August^73^) may therefore imply a higher effort of the parents, and/or a reduction of breeding success. Breeding dispersal is normally increased after the brood failed^52^. Both mechanisms, increasing the number of second clutches or deteriorating breeding conditions, may lead to a higher energy investment in reproduction at the cost of allocating energy to self-maintenance which is paid by lower survival^74,75^. An increase in the number of clutches or a reduction of nestling food availability may affect energy expenditure more in females than in males, because the energy invested in the brood may be higher for the former, and/or because the proportion of non-breeders may be higher among males compared to females.

To summarize, we currently do not know why female annual apparent survival is negatively affected by warm and dry summer conditions. However, our results indicate that weather potentially affects apparent survival of males and females differently, which may be either via differences in direct physiological effects, via food resources or via the balance of energy allocation to reproduction and self-maintenance.

Under future climate change scenarios for the Mediterranean region, summers are projected to become warmer and drier^76^, which, according to this study, could potentially lead to an increase in snowfinch female dispersal and/or a decrease in female survival. It remains uncertain whether reproductive output can be increased to compensate for a reduced survival or whether immigration from the Alps or the Pyrenees may compensate for an increased emigration. We do not expect an increase in reproduction in the future, because extreme weather events are predicted to become more frequent due to climatic change^10^, and therefore, the risk of losing a brood due to stochastic events may also increase. How strongly the populations in the Apennines are genetically connected to the ones in other mountain regions is topic of current research projects.

There is general evidence that negative population trends of cold adapted species are due to habitat loss caused by global warming^77,78^. Climate change induced habitat loss is also expected^31^ and has already been observed^26,28^ for the snowfinch. The expected decrease in female apparent survival with global warming constitutes an additional threat to this species making its future look critical. Similar threats may potentially also affect other cold-adapted species. The different response in apparent survival to climatic variables between the sexes shown in our study indicates that the mechanisms by which climate change impacts on the species demography may be complex. High quality data on demographic parameters (including breeding success, natal and breeding dispersal) from different populations of different species living at high elevations are urgently needed in order to take effective measures for counteracting the negative population trends^9,79,80^.

## Methods

### Study site and the capture-recapture data set

From June 2003 to June 2017, 671 snowfinches were caught in the Apennines, within the Gran Sasso and Monti della Laga National Park, Italy, specifically within an area of 3 km^2^ around Campo Imperatore (42°27 N, 13°34 E, 2200 m asl, see^48^). Birds were captured all year round, using mist nets and nest traps (Table 3). Number of days with snowfinch capturing ranged between 41 and 55 per year. On average, 48 field days took place between April and October, and 4 between November and March. The positioning and length of nets used for trapping, and the time spent trapping per day could not be standardised because of the highly variable spatial behaviour of the birds and the variable weather conditions.

**Table 3:** Monthly distribution of the first captures (total 671 individuals), the proportions of these birds recaptured at least once later, and the monthly distribution of the 211 recaptures between June 2003 and June 2017 (total 15 years).

Month	First captures	Proportion recaptured later	Recaptures
January	21	0.38	15
February	13	0.08	12
March	14	0.21	11
April	14	0.64	15
Mai	67	0.31	14
June	165	0.21	61
July	141	0.22	35
August	184	0.13	34
September	24	0.04	6
October	18	0.17	4
November	7	0	0
December	10	0.20	4

Snowfinches were marked with individual metal rings and, if possible, their age and sex were identified according to Strinella (2013)^59^. Of the 671 individuals captured, 101 were marked as nestlings and 570 as fully grown individuals. Almost a quarter of the individuals (157 individuals) were identified as males, 104 as females, whereas for 410 individuals (61%) sex could not be identified (Table 4). Of the 671 marked individuals, 138 were later recaptured between 1 and 6 times.

**Table 4:** Number of individuals captured in each year of the study period depicted for adult males, adult females and individuals of which the sex was not identified (mostly first year birds).

Year	Males	Females	Not-identified	Total	p Males	p Females
2003	11	9	24	44	—	—
2004	29	10	23	62	0.41 (0.14–0.77)	0.61 (0.09–0.98)
2005	32	12	48	92	0.76 (0.46–0.95)	0.65 (0.16–0.98)
2006	23	13	98	134	0.52 (0.26–0.81)	0.50 (0.08–0.97)
2007	20	6	27	53	0.75 (0.37–0.99)	0.13 (0.02–0.52)
2008	23	10	71	104	0.42 (0.19–0.77)	0.10 (0.00–0.42)
2009	7	4	14	25	0.13 (0.02–0.41)	0.33 (0.07–0.79)
2010	11	12	42	65	0.21 (0.05–0.62)	0.20 (0.03–0.68)
2011	7	7	19	33	0.33 (0.09–0.76)	0.37 (0.10–0.83)
2012	14	8	13	35	0.24 (0.04–0.67)	0.19 (0.03–0.62)
2013	1	2	4	7	0.14 (0.02–0.58)	0.10 (0.00–0.69)
2014	14	18	24	56	0.27 (0.04–0.74)	0.48 (0.11–0.95)
2015	15	11	18	44	0.45 (0.17–0.78)	0.22 (0.07–0.50)
2016	5	2	0	7	0.62 (0.23–0.97)	0.09 (0.01–0.34)
2017	1	4	2	7	0.42 (0.06–0.93)	0.71 (0.20–0.99)

The estimated annual recapture probabilities for each sex are given in the last two columns together with their 95% compatibility intervals.

Bird capturing and marking was authorised by the Institute for Environmental Protection and Research ISPRA (ES, licence CNI ISPRA no. 0114). Capturing and marking were carried out in accordance with guidelines and regulations of ISPRA.

### Weather data

We obtained data on daily minimum and maximum temperatures (°C) and precipitation (mm per day) from two local weather stations (Assergi: 42°24′N 13°30′E, 992 m asl; and Castel del Monte: 2°22′N 13°43′E, 1346 m asl; Ufficio Idrografico e Mareografico Regione Abruzzo) for the years 2003 to 2017. Daily minimum and maximum temperature were highly correlated (Pearson’s correlation r = 0.93). We used the average between the minimum and maximum temperature of both stations as a measure of average daily temperature that is sensitive to extreme temperature values. Daily precipitation was summed over the two stations in order to obtain a measure of precipitation in the study area. We then averaged daily temperature and precipitation over the summer months (June to September) and over the winter months (November to March) for each year. These four weather variables were used to predict annual apparent survival (from summer to summer).

**Figure 4:**
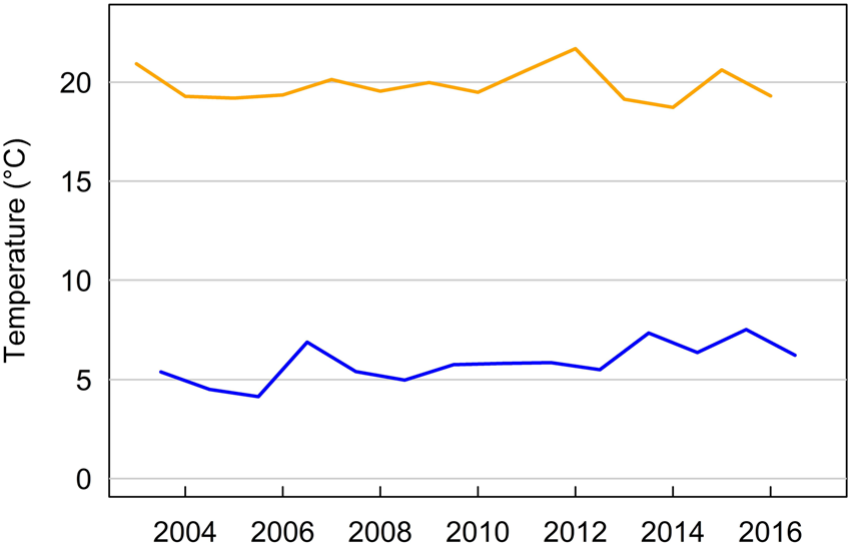
Average summer and winter temperature for each year of the study period. Summer temperature is the average temperature for the months June to September, winter temperature is the average between November and March.

Precipitation in winter correlated positively with precipitation in summer (Pearson’s correlation r = 0.50). In summer, temperature correlated negatively with precipitation (r = −0.39). Weak positive correlations existed between winter temperature and precipitation in summer (r = 0.27), and precipitation in winter (r = 0.15), respectively. All other correlations were weaker than 0.1. Over the course of the study period, average summer temperature did not show any trend, whereas average winter temperature showed a weak positive trend (Fig. 4).

We did not consider weather variables during the breeding season because most birds were captured during or shortly after the breeding season (Table 3). Consequently, the length an individual is exposed to spring conditions during its first year after marking depends on the date of marking. We only included weather variables that could unambiguously be assigned to one summer to summer interval.

### Survival models

#### General model structure

We used mark-recapture models^81–84^ that we applied to two different temporal aggregations of the mark-recapture data set. The first analysis aimed at measuring average annual apparent survival, and investigating correlations between weather variables and annual apparent survival. In the second analysis, we described seasonal patterns of apparent survival probabilities. The general model structures in both analyses were equal but they differed in the length of the time intervals (years vs. 4-months periods) and the predictors for survival (see below). For the first analysis, we aggregated the data in annual time intervals (1st January – 31st December; mean capture date within this interval is 30th June). For the second analyses, four-month time intervals were used. For the annual data, time interval $t$ was one year (of 15 years in total), and for the seasonal data, time interval $t$ was four months (of 43 4-months periods or “seasons” in total).

The observations ${y}_{it}$, an indicator of whether individual $i$ was recaptured during time interval $t$, were modelled as a Bernoulli variable conditional on the latent state of the individual birds ${z}_{it}$ (0 = dead or permanently emigrated, 1 = alive and at the study site). The probability $P({y}_{it}\mathrm{=1)}$ is the product of the probability that an alive individual is recaptured, ${p}_{it}$, and the state of the bird ${z}_{it}$. Thus, a dead or permanently emigrated bird cannot be recaptured, whereas for a bird alive during time interval $t$ the recapture probability equals ${p}_{it}$:

$${y}_{it} \sim Bernoulli({z}_{it}{p}_{it})$$

The latent state variable ${z}_{it}$ is a Markovian variable with the state at time $t$ being dependent on the state at time $t-1$ and the apparent survival probability ${\Phi }_{it}$:

$${z}_{it} \sim Bernoulli({z}_{it-1}{\Phi }_{it})$$

We use the term “apparent survival” to indicate that the parameter $\Phi$ is a product of site fidelity and survival. Thus, individuals that permanently emigrated from the study area cannot be distinguished from dead individuals.

In both models, the parameters $\Phi$ and $p$ were modelled as sex-specific. However, for 61% of the individuals, sex could not be identified, i.e. sex was missing. Ignoring the individuals with missing sex would most likely lead to a bias because they were not missing at random. The probability that sex can be identified is increasing with age and most likely differs between sexes. Further, in our data, the probability that sex could be identified varied across the study period because different methods (genetics, plumage, breeding patch) were used in different years, and sex identification literature became available during the study period^59^. As a consequence, we cannot use our data to estimate the sex-specific probability of identifying the sex of an individual^85^. However, we can include the missing sexes using a mixture model structure similarly to Pledger (2000)^86^ who introduced a mixture model for unknown classes. In our case, for part of the individuals, the class (sex) was known. We imputed the sex assignment for non-identified individuals using a categorical distribution with a uniform $Beta\mathrm{(1,1)}$ distribution for the probability of being a male ${q}_{i}\mathrm{[1]}$:

$$Se{x}_{i} \sim Categorical({{\bf{q}}}_{i})$$

where, for every non-identified individual, ${{\bf{q}}}_{i}$ is a vector of length 2, containing the probability of being a male and a female, respectively. The sex of each non-identified individual was therefore assumed to be male or female with probability ${q}_{i}\mathrm{[1]}$ and ${q}_{i}\mathrm{[2]}=1-{q}_{i}\mathrm{[1]}$, respectively. A uniform distribution between 0 and 1 was assumed for ${q}_{i}\mathrm{[1]}$. In this way, no specific sex was assigned to these individuals, but their data was used for the survival estimates preventing them to be overestimated. Indeed, the posterior distributions of the ${q}_{i}\mathrm{[1]}$ were close to a uniform distribution in all models. Therefore, we do not present them in the results.

In addition, we fitted all models without the mixture structure to a reduced data set including only individuals with identified sex and only the re-captures after their sex could first be ascertained^87^. Except for 5 individuals, all individuals were adult when their sex was ascertained. These 5 individuals were excluded from the analyses on the reduced data set. In such a reduced data set, individuals that show clear sex-specific characteristics and that are strong enough to live long will be over-represented. Consequently, the results may not be representative for the snowfinch population in the Apennines. On the other hand, also the full data set may not be a random sample of individuals because inexpert or high active individuals are more likely to be captured by mist-nets than experienced or less active individuals^88,89^. Therefore, we present the results from the analyses of both the full and reduced data sets.

#### Annual apparent survival models

We used seven different models for annual apparent survival that differed in their temporal structure of apparent survival (Table 1). In the first model, we assumed constant apparent survival over time, but included different apparent survival for age and sex classes (3 levels: first year birds, adult males and adult females):

Model 1: $${\Phi }_{it}={a}_{t,age\mathrm{}.sex[it]}$$

In the second model, we included a sex-specific random year effect

Model 2a: $$logit({\Phi }_{it})=a{0}_{age\mathrm{}.sex[it]}+{\gamma }_{sex[i]t}$$ with $${\gamma }_{sex[i]t} \sim Normal\mathrm{(0,}\sigma )$$.

The third model is similar to model 2a but it includes for each age and sex class a separate apparent survival for the first year after first capture (first occasion). It thus estimates for both sexes two adult apparent survival, one during the first year after the first capture and one during the second and later years after the first capture. Because juveniles become adults after one year, the models include only one apparent survival for juveniles.

Model 2b: $logit({\Phi }_{it})=a{0}_{age\mathrm{}.sex[it],firstoccasion[it]}+{\gamma }_{sex[i]t}$ with ${\gamma }_{sex[i]t} \sim Normal\mathrm{(0,}\sigma )$, where the variable firstoccasion contains a 1 for the first occasion and a 2 for later occasions.

In the following four models, we modelled annual apparent survival to be linearly related to average summer and average winter temperature (summertemp, wintertemp, models 3a, 3b, 23b, and 4). In the last model (model 4), we also included precipitation (summerprec, winterprec) as predictors. We estimated different effects of temperature and precipitation on apparent survival for juveniles, adult males and adult females:

Model 3a: $$logit({\Phi }_{it})=a{0}_{age\mathrm{}.sex[it]}+a{1}_{age\mathrm{}.sex[it]}summertem{p}_{t}+a{2}_{age\mathrm{}.sex[it]}wintertem{p}_{t}$$

Model 3b was similar to model 3a but included separate apparent survival and separate correlations between temperature and apparent survival during the first year after first capture and during the second or later years after the first capture.

Model 3b: $$logit({\Phi }_{it})=a{0}_{age\mathrm{}.sex[it],firstoccasion[it]}+a{1}_{age\mathrm{}.sex[it],firstoccasion[it]}summertem{p}_{t}+a{2}_{age\mathrm{}.sex[it],firstoccasion[it]}$$ $$wintertem{p}_{t}$$

Model23b combines the random year structure of model 2, the linear relationship with summer and winter temperature of model 3, and it also includes separate apparent survival for the first and later years after the first capture. However, in model 3b the correlations with temperature variables separately for first and later years after the first captures could not be estimated well (low sample size). Therefore, in model 23b we estimated only one correlation between apparent survival and each of the temperature variables and assumed that this correlation was the same for first and later years after the first capture.

Model 23b: $$logit({\Phi }_{it})=a{0}_{age\mathrm{}.sex[it],firstoccasion[it]}+a{1}_{age\mathrm{}.sex[it]}summertem{p}_{t}+a{2}_{age\mathrm{}.sex[it]}wintertem{p}_{t}+{\gamma }_{sex[i]t}$$ with $${\gamma }_{sex[i]t} \sim Normal\mathrm{(0,}\sigma )$$

In the last model, we included summer and winter temperature and summer and winter precipitation as predictors for apparent survival.

Model 4: $$logit({\Phi }_{it})=a{0}_{age\mathrm{}.sex[it]}+a{1}_{age\mathrm{}.sex[it]}summertem{p}_{t}+a{2}_{age\mathrm{}.sex[it]}wintertem{p}_{t}+a{3}_{age\mathrm{}.sex[it]}summerpre{c}_{t}+a{4}_{age\mathrm{}.sex[it]}winterpre{c}_{t}$$

In all models, annual recapture probability was modelled for each year and sex independently: ${p}_{it}=b{0}_{t,sex[it]}$. Because all individuals were at least one year old when they can be recaptured for the first time, we did not include age as a predictor for recapture probability.

Uniform prior distributions were used for all parameters with a parameter space limited to values between 0 and 1 for probabilities. A normal distribution with a mean of 0 and a standard deviation of 1.5 was used for the intercept $a0$, and for $a1$, $a2$, $a3$, and $a4$ a standard deviation of 3 was used.

#### Seasonal survival model

We assumed that four-month survival differed between age and sex classes (juveniles, adult male, adult female), and seasons (winter: December – March, breeding: April – July, summer: August â€“ November), ${\Phi }_{it}={a}_{sex\mathrm{}.age[i],season[t]}$. Independent, and slightly informative prior distributions ${a}_{sex\mathrm{}.age[i],season[t]} \sim Beta\mathrm{(3.6,1.2)}$ were used. This prior gives 95% of the mass to values between 0.33 and 0.99 and has a median of 0.79. An average survival of 0.79 over 4 months corresponds to an annual survival of 0.49. By choosing a prior distribution with a mean corresponding to approximately the overall mean of the data we make sure that estimates for specific seasons deviating from the overall mean show information that is inherent to the data. Using a uniform prior, $Beta\mathrm{(1,1)}$, with a mean of 0.5 would result in estimates close to 0.5 for seasons with a small sample size, i.e. during winter, which would bias the conclusions on seasonal differences in seasonal survival. Recapture probability was assumed to depend on season, sex and year using the logit link function and assigning a normal distribution to the year effects:

$$logit({p}_{it})=b{0}_{season[t],sex[i]}+{\gamma }_{y}ear[t]\,\mathrm{where}\,{\gamma }_{y}ear[t] \sim Normal\mathrm{(0,}\sigma )$$

Independent normal prior distributions were specified for the average logit-transformed recapture probabilities,

$$b{0}_{season[t],sex[i]} \sim Normal\mathrm{(0,1.5)}.$$

#### Model fitting and predictive model checking

We used Hamiltonian Monte Carlo as implemented in Stan^90^ to fit the models to the data. We simulated 4 Markov chains of length 2000 and used the second half of each chain for the description of the posterior distributions of the model parameters.

Convergence and mixing of the Markov chains were assessed by the metrics and diagnostic plots provided by *rstan*^91^ and *shinystan*^92^ packages, i.e. no divergent transition, number of effective samples above 1000, Monte Carlo errors below 10%, and R-hat value below 1.01.

In order to assess the goodness of fit, we used R 3.6.1^93^ to simulate from the model 1000 times new capture histories for each individual in the data. For every draw we used another set of parameter values from the simulated joint posterior distribution of the model parameters (that was generated by Hamiltonian Monte Carlo in Stan, as described above). These 1000 new data sets look like the model “thinks” the data should look like^38^. For every new data set, we extracted the number of individuals captured exactly once and the number of individuals captured at least three times. We compared these two statistics between the 1000 new data sets and the observed data.

## Supplementary information

Supplementary information.

## Data Availability

Data archived on Dryad (10.5061/dryad.6wwpzgmtt).
