# Supplementary material for: Potential sex-dependent effects of weather on apparent survival of a high-elevation specialist
Source: Sci Rep. 2020 May 20;10:8386. doi: 10.1038/s41598-020-65017-w (PMC7239909; doi:10.1038/s41598-020-65017-w)
Supplement: Supplementary file 1 — Supplementary information. [file 41598_2020_65017_MOESM1_ESM.docx]

Supplementary material: Tables S1-S2 and Stan code of the models

**Potential sex-dependent effects of weather on apparent survival of a high-elevation specialist**

**Eliseo Strinella**^1^**, Davide Scridel**^2,3^**, Mattia Brambilla**^2,4^**, Christian Schano**^5,6^**, and Fränzi Korner-Nievergelt**^5,*^

1Reparto Carabinieri Biodiversit `a L’Aquila, Italy

2Museo delle Scienze di Trento (MUSE), Sezione Zoologia dei Vertebrati, Corso del Lavoro e della Scienza 3,

38122 Trento, Italy

3Ente Parco Naturale Paneveggio Pale di San Martino, loc. Castelpietra, 2-Tonadico, Trento, Italy

4Fondazione Lombardia per l’Ambiente, Largo 10 luglio 1976 1, I-20822 Seveso (MB), Italy

5Swiss Ornithological Institute, Seerose 1, CH – 6204 Sempach, Switzerland

6University of Zurich, Department of Evolutionary Biology and Environmental Studies, Winterthurerstrasse 190, CH

– 8057 Zurich, Switzerland

*fraenzi.korner@vogelwarte.ch

Table S1. Results from posterior predictive model checking of the 6 models for estimating annual apparent survival fitted to the full and reduced data sets. Two statistics were used: The number of individuals captured only once and the number of individuals captured at least three times. In the full data set, we measured the fit for the data with known and unknown sexes separately. For the expected values the median and the 99% interval of the posterior predictive distribution are given. The Bayesian p-value is the proportion of simulated replicated data from the model that had a higher statistics than the observed data. Bayesian p-values close to 0 or 1 indicate a lack of fit. Such cases are marked by an exclamation mark (!). 1000 replicated data sets (i.e., data sets simulated from the model) were used. Marked in grey are the models with the best fit for the specific data set.

| ***Full data set*** | | | | | | |
| --- | --- | --- | --- | --- | --- | --- |
| **Model** | **Nr inds 1 capture obs** | **Nr inds 1 capture expected** | **Bayesian p-value**  **Nr inds 1 capture** | **Nr inds captured 3+ times obs** | **Nr inds captured 3+ times expected** | **Bayesian p-value**  **Nr inds captured 3+ times** |
| 1 | ident: 209  non-ident: 389 | 189 (162 – 215)  355 (321 – 377) | 0.02  <0.001 (!) | 16  2 | 21 (8 – 39)  15 (4-30) | 0.82  >0.999 (!) |
| 2a | ident: 209  non-ident: 389 | 185 (161 – 208)  350 (316 – 374) | 0.005  <0.001 (!) | 16  2 | 23 (10 – 38)  17 (5 – 33) | 0.86  >0.999 (!) |
| 2b | ident: 209  non-ident: 389 | 207 (182 – 229)  360 (327 – 380) | 0.40  <0.001 (!) | 16  2 | 21 (9 – 36)  18 (6 – 33) | 0.79  >0.999 (!) |
| 3a | ident: 209  non-ident: 389 | 182 (155 – 207)  343 (301 – 371) | 0.01  <0.001 (!) | 16  2 | 25 (11 – 44)  19 (8 – 37) | 0.91  >0.999 (!) |
| 3b | ident: 209  non-ident: 389 | 206 (161 – 231)  358 (304 – 381) | 0.40  <0.001 (!) | 16  2 | 12 (2 – 46)  10 (1 – 42) | 0.30  0.98 |
| 23b | ident: 209  non-ident: 389 | 202 (173 - 230)  353 (312 - 378) | 0.25  <0.001 (!) | 16  2 | 22 (10 - 42)  20 (8 - 38) | 0.85  >0.999 (!) |
| 4 | ident: 209  non-ident: 389 | 176 (143 – 203)  343 (312 – 366) | <0.001 (!)  <0.001 (!) | 16  2 | 27 (13 – 51)  21 (9 – 39) | 0.96  >0.999 (!) |
| ***Reduced data set*** | | | | | | |
| 1 | 191 | 183 (154 – 206) | 0.16 | 17 | 18 (7 – 34) | 0.54 |
| 2a | 191 | 180 (151 – 203) | 0.11 | 17 | 19 (8 – 37) | 0.61 |
| 2b | 191 | 190 (158 – 214) | 0.42 | 17 | 20 (8 – 37) | 0.69 |
| 3a | 191 | 180 (151 – 202) | 0.11 | 17 | 19 (7 – 36) | 0.62 |
| 3b | 191 | 187 (152 – 212) | 0.33 | 17 | 22 (9 – 40) | 0.76 |
| 23b | 191 | 187 (157 - 210) | 0.34 | 17 | 21 (9 - 38) | 0.74 |
| 4 | 191 | 175 (147 – 200) | 0.06 | 17 | 21 (8 – 37) | 0.73 |

Table S2. Estimates for annual apparent survival of individuals ringed as nestlings, first year birds or adult males (m) or females (f) based on different models and two different data sets. Models 3a, 3b, 23b, and 4 contained both sex and age dependent effects of weather (summer and winter temperature and precipitation), of which only the effects for the adults are given. The models were fitted to the full data set and to a data set reduced to the individuals with identified sex only including recaptures after the sex was first identified. In addition, for the reduced data set, models 2 and 3 were expanded to estimate apparent survival separately for the first (Phi1) and later (Phi2+) years after capture. Parameters that were not included in the model are indicated with “-“.

| ***Full data set*** | | | | | | | | |
| --- | --- | --- | --- | --- | --- | --- | --- | --- |
| **Model** | **Phi nestl.** | **Phi juveniles** | **Phi males** | **Phi females** | **summer**  **temp** | **winter**  **temp** | **summer**  **precip** | **winter**  **precip** |
| 1 | 0.15 (0.01, 0.42) | 0.15 (0.03, 0.33) | 0.40 (0.32, 0.49) | 0.42 (0.33, 0.52) | - | - | - | - |
| 2a | 0.16 (0.06, 0.37) | 0.16 (0.08, 0.32) | 0.40 (0.30, 0.54) | 0.55 (0.33, 0.86) | - | - | - | - |
| 2b | 0.13 (0.05, 0.29) | 0.13 (0.06, 0.26) | Phi1: 0.26 (0.16, 0.42)  Phi2+: 0.50 (0.36, 0.68) | Phi1: 0.35 (0.16, 0.69)  Phi2+: 0.59 (0.34, 0.86) | - | - | - | - |
| 3a | 0.15 (0.04, 0.42) | 0.11 (0.05, 0.24) | 0.41 (0.32, 0.53) | 0.45 (0.31, 0.79) | m: 0.18 (-0.47, 0.92)  f: -0.78 (-1.75, 0.03) | m: -0.07 (-0.56, 0.42)  f: 0.99 (0.02, 2.66) | - | - |
| 3b | 0.12 (0.04, 0.34) | 0.09 (0.04, 0.19) | Phi1: 0.26 (0.16, 0.40)  Phi2+:0.54 (0.40, 0.71) | Phi1: 0.33 (0.14, 0.86)  Phi2+: 0.54 (0.34, 0.86) | m Phi1: 0.03 (-0.55, 0.67)  f Phi1: -1.12 (-2.53, -0.08)  m Phi2+: 0.36 (-0.75, 1.74)  f Phi2+: 0.86 (-1.42,3.93) | m Phi1: -0.17 (-0.68, 0.33)  f Phi1: 0.71 (-1.12,3.04)  m Phi2+: -0.06 (-0.75, 0.66)  f Phi2+: 0.26 (-1.07,1.87) | - | - |
| 23b | 0.12 (0.04,0.34) | 0.11 (0.04,0.26) | Phi1: 0.28 (0.16,0.55)  Phi2+: 0.52 (0.36,0.75) | Phi1: 0.38 (0.17,0.77)  Phi2+: 0.64 (0.36,0.91) | m: 0.05 (-0.79,0.98)  f: -0.85 (-2.18,0.42) | m: -0.08 (-0.76,0.63)  f: 0.37 (-1.08,1.74) | - | - |
| 4 | 0.22 (0.05, 0.64) | 0.12 (0.04, 0.36) | 0.46 (0.36, 0.77) | 0.51 (0.35, 0.84) | m: 0.67 (-0.58, 2.72)  f: -0.85 (-2.09, 0.22) | m: -0.16 (-0.86, 0.51)  f: 0.92 (-0.29, 2.75) | m: 0.54 (-0.87, 2.42)  f: 0.13 (-1.24,1.71) | m: -0.10 (-1.41,1.19)  f: -0.01 (-1.55,1.60) |
| 5 | 0.17 (007, 0.33)^*)^ | | 0.51 (0.41, 0.61) | 0.53 (0.41, 0.64) | - | - | - | - |
| ***Reduced data set*** | | | | | | | | |
| **Model** | **Phi1 males** | **Phi1 females** | **Phi or Phi2+**  **males** | **Phi or Phi2+ females** | **summer**  **temp** | **winter**  **temp** | **summer**  **precip** | **winter**  **precip** |
| 1 | - | - | 0.35 (0.27, 0.44) | 0.42 (0.32, 0.53) | - | - | - | - |
| 2a | - | - | 0.36 (0.26,0.52) | 0.43 (0.28, 0.68) | - | - | - | - |
| 2b | 0.28 (0.17, 0.47) | 0.35 (0.16, 0.64) | 0.47 (0.31,0.68) | 0.53 (0.31, 0.81) | - | - | - | - |
| 3a | - | - | 0.35 (0.27,0.45) | 0.44 (0.32, 0.60) | m: -0.17 (-0.75, 0.42)  f: -0.70 (-1.61,0.07) | m:0.08 (-0.36,0.54)  f: 0.44 (-0.26, 1.36) | - | - |
| 3b | 0.26 (0.17,0.39) | 0.33 (0.16,0.77) | 0.44 (0.29, 0.62) | 0.51 (0.30, 0.83) | first occ  m: -0.15 (-0.70,0.42)  f: -1.07 (-3.05, -0.16)  later  m: -0.16 (-1.44,1.04)  f: 0.35 (-1.68,3.36) | first occ  m: -0.22 (-0.77, 0.30)  f: -0.55 (-4.25,1.02)  later  m: 0.58 (-0.17, 1.49)  f: 0.44 (-0.87,2.12) | - | - |
| 23b | 0.29 (0.17,0.52) | 0.34 (0.14,0.69) | 0.49 (0.31,0.72) | 0.57 (0.32,0.88) | m: -0.18 (-1.11,0.68)  f: -0.72 (-1.98,0.62) | m: 0.05 (-0.71,0.84)  f: -0.02 (-1.77,1.25) | - | - |
| 4 | - | - | 0.40 (0.30, 0.56) | 0.47 (0.33, 0.76) | m: 0.04 (-0.87, 1.15)  f: -0.20 (-1.46,1.62) | m: -0.07 (-0.67, 0.57)  f: 0.02 (-1.09,1.34) | m: 0.85 (-0.57,2.48)  f: 1.24 (-0.29,3.22 ) | m: -0.19 (-1.56,1.27)  f:-0.67 (-2.31, 0.82) |
| 5 | - | - | 0.43 (0.34, 0.53) | 0.50 (0.39,0.62) | - | - | - | - |

*) Juvenile annual apparent survival is calculated as the product of apparent survival from breeding to summer/fall and from summer/fall to winter and from winter to breeding for nestlings and first year birds respectively.

Stan code of the models

**Model 1 for full data set**

data {

int<lower=2> nocc; // number of capture events

int<lower=0> nindi; // number of individuals with identified sex

int<lower=0> nindni; // number of individuals with non-identified sex

int<lower=0,upper=2> yi[nindi,nocc]; // CH[i,k]: individual i captured at k

int<lower=0,upper=nocc-1> firsti[nindi]; // year of first capture

int<lower=0,upper=2> yni[nindni,nocc]; // CH[i,k]: individual i captured at k

int<lower=0,upper=nocc-1> firstni[nindni]; // year of first capture

int<lower=1, upper=2> sex[nindi];

int<lower=1, upper=3> juvi[nindi, nocc]; // 1 = ad, 2 = fully grown first year, 3 = pulli

int<lower=1, upper=3> juvni[nindni, nocc];

}

transformed data {

int<lower=0,upper=nocc+1> lasti[nindi]; // lasti[i]: last capture for identified inds

int<lower=0,upper=nocc+1> lastni[nindni]; // lastni[i]: last capture for not identified inds

lasti = rep_array(0,nindi);

lastni = rep_array(0,nindni);

for (i in 1:nindi) {

for (k in firsti[i]:nocc) {

if (yi[i,k] == 1) {

if (k > lasti[i]) lasti[i] = k;

}

}

}

for (ii in 1:nindni) {

for (kk in firstni[ii]:nocc) {

if (yni[ii,kk] == 1) {

if (kk > lastni[ii]) lastni[ii] = kk;

}

}

}

}

parameters {

real<lower=0, upper=1> theta[nindni]; // probability of being male for non-identified individuals

real<lower=0, upper=1> b0[2,nocc-1]; // intercept of p

real<lower=0, upper=1> a0[2,3]; // intercept for phi

}

transformed parameters {

real<lower=0,upper=1>p_male[nindni,nocc]; // capture probability

real<lower=0,upper=1>p_female[nindni,nocc]; // capture probability

real<lower=0,upper=1>p[nindi,nocc]; // capture probability

real<lower=0,upper=1>phi_male[nindni,nocc-1]; // survival probability

real<lower=0,upper=1>chi_male[nindni,nocc+1]; // probability that an individual

// is never recaptured after its

// last capture

real<lower=0,upper=1>phi_female[nindni,nocc-1]; // survival probability

real<lower=0,upper=1>chi_female[nindni,nocc+1]; // probability that an individual

// is never recaptured after its

// last capture

real<lower=0,upper=1>phi[nindi,nocc-1]; // survival probability

real<lower=0,upper=1>chi[nindi,nocc+1]; // probability that an individual

// is never recaptured after its

// last capture

{

int k;

int kk;

for(ii in 1:nindi){

if (firsti[ii]>1) {

for (z in 1:(firsti[ii]-1)){

phi[ii,z] = 1;

}

}

for(tt in firsti[ii]:(nocc-1)) {

// linear predictor for phi:

phi[ii,tt] = a0[sex[ii], juvi[ii,tt]];

}

}

for(ii in 1:nindni){

if (firstni[ii]>1) {

for (z in 1:(firstni[ii]-1)){

phi_female[ii,z] = 1;

phi_male[ii,z] = 1;

}

}

for(tt in firstni[ii]:(nocc-1)) {

// linear predictor for phi:

phi_male[ii,tt] = a0[1, juvni[ii,tt]];

phi_female[ii,tt] = a0[2, juvni[ii,tt]];

}

}

for(i in 1:nindi) {

// linear predictor for p for identified individuals

for(w in 1:firsti[i]){

p[i,w] = 1;

}

for(kkk in (firsti[i]+1):nocc)

p[i,kkk] = b0[sex[i],kkk-1];

chi[i,nocc+1] = 1.0;

k = nocc;

while (k > firsti[i]) {

chi[i,k] = (1 - phi[i,k-1]) + phi[i,k-1] * (1 - p[i,k]) * chi[i,k+1];

k = k - 1;

}

if (firsti[i]>1) {

for (u in 1:(firsti[i]-1)){

chi[i,u] = 0;

}

}

chi[i,firsti[i]] = (1 - p[i,firsti[i]]) * chi[i,firsti[i]+1];

}// close definition of transformed parameters for identified individuals

for(i in 1:nindni) {

// linear predictor for p for non-identified individuals

for(w in 1:firstni[i]){

p_male[i,w] = 1;

p_female[i,w] = 1;

}

for(kkkk in (firstni[i]+1):nocc){

p_male[i,kkkk] = b0[1,kkkk-1];

p_female[i,kkkk] = b0[2,kkkk-1];

}

chi_male[i,nocc+1] = 1.0;

chi_female[i,nocc+1] = 1.0;

k = nocc;

while (k > firstni[i]) {

chi_male[i,k] = (1 - phi_male[i,k-1]) + phi_male[i,k-1] * (1 - p_male[i,k]) * chi_male[i,k+1];

chi_female[i,k] = (1 - phi_female[i,k-1]) + phi_female[i,k-1] * (1 - p_female[i,k]) * chi_female[i,k+1];

k = k - 1;

}

if (firstni[i]>1) {

for (u in 1:(firstni[i]-1)){

chi_male[i,u] = 0;

chi_female[i,u] = 0;

}

}

chi_male[i,firstni[i]] = (1 - p_male[i,firstni[i]]) * chi_male[i,firstni[i]+1];

chi_female[i,firstni[i]] = (1 - p_female[i,firstni[i]]) * chi_female[i,firstni[i]+1];

} // close definition of transformed parameters for non-identified individuals

} // close block of transformed parameters exclusive parameter declarations

} // close transformed parameters

model {

// priors

theta ~ beta(1, 1);

for (g in 1:(nocc-1)){

b0[1,g]~beta(1,1);

b0[2,g]~beta(1,1);

}

a0[1,1]~beta(1,1);

a0[1,2]~beta(1,1);

a0[2,1]~beta(1,1);

a0[2,2]~beta(1,1);

a0[1,3]~beta(1,1);

a0[2,3]~beta(1,1);

// likelihood for identified individuals

for (i in 1:nindi) {

if (lasti[i]>0) {

for (k in firsti[i]:lasti[i]) {

if(k>1) target+= (log(phi[i, k-1]));

if (yi[i,k] == 1) target+=(log(p[i,k]));

else target+=(log1m(p[i,k]));

}

}

target+=(log(chi[i,lasti[i]+1]));

}

// likelihood for non-identified individuals

for (i in 1:nindni) {

real log_like_male = 0;

real log_like_female = 0;

if (lastni[i]>0) {

for (k in firstni[i]:lastni[i]) {

if(k>1){

log_like_male += (log(phi_male[i, k-1]));

log_like_female += (log(phi_female[i, k-1]));

}

if (yni[i,k] == 1){

log_like_male+=(log(p_male[i,k]));

log_like_female+=(log(p_female[i,k]));

}

else{

log_like_male+=(log1m(p_male[i,k]));

log_like_female+=(log1m(p_female[i,k]));

}

}

}

log_like_male += (log(chi_male[i,lastni[i]+1]));

log_like_female += (log(chi_female[i,lastni[i]+1]));

target += log_mix(theta[i], log_like_male, log_like_female);

}

}

**Model 1 for reduced data set**

data {

int<lower=2> nocc; // number of capture events

int<lower=0> nind; // number of individuals with identified sex

int<lower=0,upper=2> y[nind,nocc]; // CH[i,k]: individual i captured at k

int<lower=0,upper=nocc-1> first[nind]; // year of first capture

int<lower=1, upper=2> sex[nind];

int<lower=1> year[nocc];

}

transformed data {

int<lower=0,upper=nocc+1> last[nind]; // last[i]: ind i last capture

last = rep_array(0,nind);

for (i in 1:nind) {

for (k in first[i]:nocc) {

if (y[i,k] == 1) {

if (k > last[i]) last[i] = k;

}

}

}

}

parameters {

real<lower=0, upper=1> b0[2,nocc-1]; // intercept of p

real a0[2]; // intercept for phi

}

transformed parameters {

real<lower=0,upper=1>p[nind,nocc]; // capture probability

real<lower=0,upper=1>phi[nind,nocc-1]; // survival probability

real<lower=0,upper=1>chi[nind,nocc+1]; // probability that an individual

// is never recaptured after its

// last capture

{

int k;

for(ii in 1:nind){

if (first[ii]>1) {

for (z in 1:(first[ii]-1)){

phi[ii,z] = 1;

}

}

for(tt in first[ii]:(nocc-1)) {

// linear predictor for phi:

phi[ii,tt] = inv_logit(a0[sex[ii]]);

}

}

for(i in 1:nind) {

// linear predictor for p for identified individuals

for(w in 1:first[i]){

p[i,w] = 1;

}

for(kkk in (first[i]+1):nocc)

p[i,kkk] = b0[sex[i],year[kkk-1]];

chi[i,nocc+1] = 1.0;

k = nocc;

while (k > first[i]) {

chi[i,k] = (1 - phi[i,k-1]) + phi[i,k-1] * (1 - p[i,k]) * chi[i,k+1];

k = k - 1;

}

if (first[i]>1) {

for (u in 1:(first[i]-1)){

chi[i,u] = 0;

}

}

chi[i,first[i]] = (1 - p[i,first[i]]) * chi[i,first[i]+1];

}// close definition of transformed parameters for identified individuals

} // close block of transformed parameters exclusive parameter declarations

} // close transformed parameters

model {

// priors

for (g in 1:(nocc-1)){

b0[1,g]~beta(1,1);

b0[2,g]~beta(1,1);

}

a0[1]~normal(0,1.5);

a0[2]~normal(0,1.5);

// likelihood for identified individuals

for (i in 1:nind) {

if (last[i]>0) {

for (k in first[i]:last[i]) {

if(k>1) target+= (log(phi[i, k-1]));

if (y[i,k] == 1) target+=(log(p[i,k]));

else target+=(log1m(p[i,k]));

}

}

target+=(log(chi[i,last[i]+1]));

}

}

**Model 2a for full data set**

data {

int<lower=2> nocc; // number of capture events

int<lower=0> nindi; // number of individuals with identified sex

int<lower=0> nindni; // number of individuals with non-identified sex

int<lower=0,upper=2> yi[nindi,nocc]; // CH[i,k]: individual i captured at k

int<lower=0,upper=nocc-1> firsti[nindi]; // year of first capture

int<lower=0,upper=2> yni[nindni,nocc]; // CH[i,k]: individual i captured at k

int<lower=0,upper=nocc-1> firstni[nindni]; // year of first capture

int<lower=1, upper=2> sex[nindi];

int<lower=1, upper=3> juvi[nindi, nocc]; // 1 = ad, 2 = fully grown first year, 3 = pulli

int<lower=1, upper=3> juvni[nindni, nocc];

}

transformed data {

int<lower=0,upper=nocc+1> lasti[nindi]; // last[i]: ind i last capture

int<lower=0,upper=nocc+1> lastni[nindni]; // last[i]: ind i last capture

lasti = rep_array(0,nindi);

lastni = rep_array(0,nindni);

for (i in 1:nindi) {

for (k in firsti[i]:nocc) {

if (yi[i,k] == 1) {

if (k > lasti[i]) lasti[i] = k;

}

}

}

for (ii in 1:nindni) {

for (kk in firstni[ii]:nocc) {

if (yni[ii,kk] == 1) {

if (kk > lastni[ii]) lastni[ii] = kk;

}

}

}

}

parameters {

real<lower=0, upper=1> theta[nindni]; // probability of being male for non-identified individuals

real<lower=0, upper=1> b0[2,nocc-1]; // intercept of p

real a0[2,3]; // intercept for phi

real<lower=0> sigma[2];

real yeareff[2,nocc-1];

}

transformed parameters {

real<lower=0,upper=1>p_male[nindni,nocc]; // capture probability

real<lower=0,upper=1>p_female[nindni,nocc]; // capture probability

real<lower=0,upper=1>p[nindi,nocc]; // capture probability

real<lower=0,upper=1>phi_male[nindni,nocc-1]; // survival probability

real<lower=0,upper=1>chi_male[nindni,nocc+1]; // probability that an individual

// is never recaptured after its

// last capture

real<lower=0,upper=1>phi_female[nindni,nocc-1]; // survival probability

real<lower=0,upper=1>chi_female[nindni,nocc+1]; // probability that an individual

// is never recaptured after its

// last capture

real<lower=0,upper=1>phi[nindi,nocc-1]; // survival probability

real<lower=0,upper=1>chi[nindi,nocc+1]; // probability that an individual

// is never recaptured after its

// last capture

{

int k;

int kk;

for(ii in 1:nindi){

if (firsti[ii]>1) {

for (z in 1:(firsti[ii]-1)){

phi[ii,z] = 1;

}

}

for(tt in firsti[ii]:(nocc-1)) {

// linear predictor for phi:

phi[ii,tt] = inv_logit(a0[sex[ii], juvi[ii,tt]] + sigma[sex[ii]]*yeareff[sex[ii],tt]);

}

}

for(ii in 1:nindni){

if (firstni[ii]>1) {

for (z in 1:(firstni[ii]-1)){

phi_female[ii,z] = 1;

phi_male[ii,z] = 1;

}

}

for(tt in firstni[ii]:(nocc-1)) {

// linear predictor for phi:

phi_male[ii,tt] = inv_logit(a0[1, juvni[ii,tt]] + sigma[1]*yeareff[1,tt]) ;

phi_female[ii,tt] = inv_logit(a0[2, juvni[ii,tt]]+ sigma[2]*yeareff[2,tt]);

}

}

for(i in 1:nindi) {

// linear predictor for p for identified individuals

for(w in 1:firsti[i]){

p[i,w] = 1;

}

for(kkk in (firsti[i]+1):nocc)

p[i,kkk] = b0[sex[i],kkk-1];

chi[i,nocc+1] = 1.0;

k = nocc;

while (k > firsti[i]) {

chi[i,k] = (1 - phi[i,k-1]) + phi[i,k-1] * (1 - p[i,k]) * chi[i,k+1];

k = k - 1;

}

if (firsti[i]>1) {

for (u in 1:(firsti[i]-1)){

chi[i,u] = 0;

}

}

chi[i,firsti[i]] = (1 - p[i,firsti[i]]) * chi[i,firsti[i]+1];

}// close definition of transformed parameters for identified individuals

for(i in 1:nindni) {

// linear predictor for p for non-identified individuals

for(w in 1:firstni[i]){

p_male[i,w] = 1;

p_female[i,w] = 1;

}

for(kkkk in (firstni[i]+1):nocc){

p_male[i,kkkk] = b0[1,kkkk-1];

p_female[i,kkkk] = b0[2,kkkk-1];

}

chi_male[i,nocc+1] = 1.0;

chi_female[i,nocc+1] = 1.0;

k = nocc;

while (k > firstni[i]) {

chi_male[i,k] = (1 - phi_male[i,k-1]) + phi_male[i,k-1] * (1 - p_male[i,k]) * chi_male[i,k+1];

chi_female[i,k] = (1 - phi_female[i,k-1]) + phi_female[i,k-1] * (1 - p_female[i,k]) * chi_female[i,k+1];

k = k - 1;

}

if (firstni[i]>1) {

for (u in 1:(firstni[i]-1)){

chi_male[i,u] = 0;

chi_female[i,u] = 0;

}

}

chi_male[i,firstni[i]] = (1 - p_male[i,firstni[i]]) * chi_male[i,firstni[i]+1];

chi_female[i,firstni[i]] = (1 - p_female[i,firstni[i]]) * chi_female[i,firstni[i]+1];

} // close definition of transformed parameters for non-identified individuals

} // close block of transformed parameters exclusive parameter declarations

} // close transformed parameters

model {

// priors

theta ~ beta(1, 1);

for (g in 1:(nocc-1)){

b0[1,g]~beta(1,1);

b0[2,g]~beta(1,1);

yeareff[1,g] ~ normal(0,1);

yeareff[2,g] ~ normal(0,1);

}

a0[1,1]~normal(0,1.5);

a0[1,2]~normal(0,1.5);

a0[1,3]~normal(0,1.5);

a0[2,1]~normal(0,1.5);

a0[2,2]~normal(0,1.5);

a0[2,3]~normal(0,1.5);

sigma~cauchy(0,3);

// likelihood for identified individuals

for (i in 1:nindi) {

if (lasti[i]>0) {

for (k in firsti[i]:lasti[i]) {

if(k>1) target+= (log(phi[i, k-1]));

if (yi[i,k] == 1) target+=(log(p[i,k]));

else target+=(log1m(p[i,k]));

}

}

target+=(log(chi[i,lasti[i]+1]));

}

// likelihood for non-identified individuals

for (i in 1:nindni) {

real log_like_male = 0;

real log_like_female = 0;

if (lastni[i]>0) {

for (k in firstni[i]:lastni[i]) {

if(k>1){

log_like_male += (log(phi_male[i, k-1]));

log_like_female += (log(phi_female[i, k-1]));

}

if (yni[i,k] == 1){

log_like_male+=(log(p_male[i,k]));

log_like_female+=(log(p_female[i,k]));

}

else{

log_like_male+=(log1m(p_male[i,k]));

log_like_female+=(log1m(p_female[i,k]));

}

}

}

log_like_male += (log(chi_male[i,lastni[i]+1]));

log_like_female += (log(chi_female[i,lastni[i]+1]));

target += log_mix(theta[i], log_like_male, log_like_female);

}

}

**Model 2a for reduced data set**

data {

int<lower=2> nocc; // number of capture events

int<lower=0> nind; // number of individuals with identified sex

int<lower=0,upper=2> y[nind,nocc]; // CH[i,k]: individual i captured at k

int<lower=0,upper=nocc-1> first[nind]; // year of first capture

int<lower=1, upper=2> sex[nind];

int<lower=1> year[nocc];

}

transformed data {

int<lower=0,upper=nocc+1> last[nind]; // last[i]: ind i last capture

last = rep_array(0,nind);

for (i in 1:nind) {

for (k in first[i]:nocc) {

if (y[i,k] == 1) {

if (k > last[i]) last[i] = k;

}

}

}

}

parameters {

real<lower=0, upper=1> b0[2,nocc-1]; // intercept of p

real a0[2]; // intercept for phi

real<lower=0> sigma[2];

real yeareff[2, nocc-1];

}

transformed parameters {

real<lower=0,upper=1>p[nind,nocc]; // capture probability

real<lower=0,upper=1>phi[nind,nocc-1]; // survival probability

real<lower=0,upper=1>chi[nind,nocc+1]; // probability that an individual

// is never recaptured after its

// last capture

{

int k;

for(ii in 1:nind){

if (first[ii]>1) {

for (z in 1:(first[ii]-1)){

phi[ii,z] = 1;

}

}

for(tt in first[ii]:(nocc-1)) {

// linear predictor for phi:

phi[ii,tt] = inv_logit(a0[sex[ii]] + sigma[sex[ii]]*yeareff[sex[ii],tt]);

}

}

for(i in 1:nind) {

// linear predictor for p for identified individuals

for(w in 1:first[i]){

p[i,w] = 1;

}

for(kk in (first[i]+1):nocc)

p[i,kk] = b0[sex[i],year[kk-1]];

chi[i,nocc+1] = 1.0;

k = nocc;

while (k > first[i]) {

chi[i,k] = (1 - phi[i,k-1]) + phi[i,k-1] * (1 - p[i,k]) * chi[i,k+1];

k = k - 1;

}

if (first[i]>1) {

for (u in 1:(first[i]-1)){

chi[i,u] = 0;

}

}

chi[i,first[i]] = (1 - p[i,first[i]]) * chi[i,first[i]+1];

}// close definition of transformed parameters for identified individuals

} // close block of transformed parameters exclusive parameter declarations

} // close transformed parameters

model {

// priors

for (g in 1:(nocc-1)){

b0[1,g]~beta(1,1);

b0[2,g]~beta(1,1);

yeareff[1, g]~normal(0,1);

yeareff[2, g]~normal(0,1);

}

a0[1]~normal(0,1.5);

a0[2]~normal(0,1.5);

sigma~cauchy(0,3);

// likelihood

for (i in 1:nind) {

if (last[i]>0) {

for (k in first[i]:last[i]) {

if(k>1) target+= (log(phi[i, k-1]));

if (y[i,k] == 1) target+=(log(p[i,k]));

else target+=(log1m(p[i,k]));

}

}

target+=(log(chi[i,last[i]+1]));

}

}

**Model 2b for full data set**

data {

int<lower=2> nocc; // number of capture events

int<lower=0> nindi; // number of individuals with identified sex

int<lower=0> nindni; // number of individuals with non-identified sex

int<lower=0,upper=2> yi[nindi,nocc]; // CH[i,k]: individual i captured at k

int<lower=0,upper=nocc-1> firsti[nindi]; // year of first capture

int<lower=0,upper=2> yni[nindni,nocc]; // CH[i,k]: individual i captured at k

int<lower=0,upper=nocc-1> firstni[nindni]; // year of first capture

int<lower=1, upper=2> sex[nindi];

int<lower=1, upper=3> juvi[nindi, nocc]; // 1 = ad, 2 = fully grown first year, 3 = pulli

int<lower=1, upper=3> juvni[nindni, nocc];

int<lower=1, upper=2> transientsi[nindi, nocc];

int<lower=1, upper=2> transientsni[nindni, nocc];

}

transformed data {

int<lower=0,upper=nocc+1> lasti[nindi]; // lasti[i]: last capture

int<lower=0,upper=nocc+1> lastni[nindni]; // lastni[i]: last capture

lasti = rep_array(0,nindi);

lastni = rep_array(0,nindni);

for (i in 1:nindi) {

for (k in firsti[i]:nocc) {

if (yi[i,k] == 1) {

if (k > lasti[i]) lasti[i] = k;

}

}

}

for (ii in 1:nindni) {

for (kk in firstni[ii]:nocc) {

if (yni[ii,kk] == 1) {

if (kk > lastni[ii]) lastni[ii] = kk;

}

}

}

}

parameters {

real<lower=0, upper=1> theta[nindni]; // probability of being male for non-identified individuals

real<lower=0, upper=1> b0[2,nocc-1]; // intercept of p

real a0[2,3,2]; // intercept for phi

real<lower=0> sigma[2];

real yeareff[2,nocc-1];

}

transformed parameters {

real<lower=0,upper=1>p_male[nindni,nocc]; // capture probability

real<lower=0,upper=1>p_female[nindni,nocc]; // capture probability

real<lower=0,upper=1>p[nindi,nocc]; // capture probability

real<lower=0,upper=1>phi_male[nindni,nocc-1]; // survival probability

real<lower=0,upper=1>chi_male[nindni,nocc+1]; // probability that an individual

// is never recaptured after its

// last capture

real<lower=0,upper=1>phi_female[nindni,nocc-1]; // survival probability

real<lower=0,upper=1>chi_female[nindni,nocc+1]; // probability that an individual

// is never recaptured after its

// last capture

real<lower=0,upper=1>phi[nindi,nocc-1]; // survival probability

real<lower=0,upper=1>chi[nindi,nocc+1]; // probability that an individual

// is never recaptured after its

// last capture

{

int k;

int kk;

for(ii in 1:nindi){

if (firsti[ii]>1) {

for (z in 1:(firsti[ii]-1)){

phi[ii,z] = 1;

}

}

for(tt in firsti[ii]:(nocc-1)) {

// linear predictor for phi:

phi[ii,tt] = inv_logit(a0[sex[ii], juvi[ii,tt], transientsi[ii,tt]] + sigma[sex[ii]]*yeareff[sex[ii],tt]);

}

}

for(ii in 1:nindni){

if (firstni[ii]>1) {

for (z in 1:(firstni[ii]-1)){

phi_female[ii,z] = 1;

phi_male[ii,z] = 1;

}

}

for(tt in firstni[ii]:(nocc-1)) {

// linear predictor for phi:

phi_male[ii,tt] = inv_logit(a0[1, juvni[ii,tt], transientsni[ii,tt]] + sigma[1]*yeareff[1,tt]) ;

phi_female[ii,tt] = inv_logit(a0[2, juvni[ii,tt], transientsni[ii,tt]]+ sigma[2]*yeareff[2,tt]);

}

}

for(i in 1:nindi) {

// linear predictor for p for identified individuals

for(w in 1:firsti[i]){

p[i,w] = 1;

}

for(kkk in (firsti[i]+1):nocc)

p[i,kkk] = b0[sex[i],kkk-1];

chi[i,nocc+1] = 1.0;

k = nocc;

while (k > firsti[i]) {

chi[i,k] = (1 - phi[i,k-1]) + phi[i,k-1] * (1 - p[i,k]) * chi[i,k+1];

k = k - 1;

}

if (firsti[i]>1) {

for (u in 1:(firsti[i]-1)){

chi[i,u] = 0;

}

}

chi[i,firsti[i]] = (1 - p[i,firsti[i]]) * chi[i,firsti[i]+1];

}// close definition of transformed parameters for identified individuals

for(i in 1:nindni) {

// linear predictor for p for non-identified individuals

for(w in 1:firstni[i]){

p_male[i,w] = 1;

p_female[i,w] = 1;

}

for(kkkk in (firstni[i]+1):nocc){

p_male[i,kkkk] = b0[1,kkkk-1];

p_female[i,kkkk] = b0[2,kkkk-1];

}

chi_male[i,nocc+1] = 1.0;

chi_female[i,nocc+1] = 1.0;

k = nocc;

while (k > firstni[i]) {

chi_male[i,k] = (1 - phi_male[i,k-1]) + phi_male[i,k-1] * (1 - p_male[i,k]) * chi_male[i,k+1];

chi_female[i,k] = (1 - phi_female[i,k-1]) + phi_female[i,k-1] * (1 - p_female[i,k]) * chi_female[i,k+1];

k = k - 1;

}

if (firstni[i]>1) {

for (u in 1:(firstni[i]-1)){

chi_male[i,u] = 0;

chi_female[i,u] = 0;

}

}

chi_male[i,firstni[i]] = (1 - p_male[i,firstni[i]]) * chi_male[i,firstni[i]+1];

chi_female[i,firstni[i]] = (1 - p_female[i,firstni[i]]) * chi_female[i,firstni[i]+1];

} // close definition of transformed parameters for non-identified individuals

} // close block of transformed parameters exclusive parameter declarations

} // close transformed parameters

model {

// priors

theta ~ beta(1, 1);

for (g in 1:(nocc-1)){

b0[1,g]~beta(1,1);

b0[2,g]~beta(1,1);

yeareff[1,g] ~ normal(0,1);

yeareff[2,g] ~ normal(0,1);

}

a0[1,1,1]~normal(0,1.5);

a0[1,2,1]~normal(0,1.5);

a0[1,3,1]~normal(0,1.5);

a0[2,1,1]~normal(0,1.5);

a0[2,2,1]~normal(0,1.5);

a0[2,3,1]~normal(0,1.5);

a0[1,1,2]~normal(0,1.5);

a0[1,2,2]~normal(0,1.5);

a0[1,3,2]~normal(0,1.5);

a0[2,1,2]~normal(0,1.5);

a0[2,2,2]~normal(0,1.5);

a0[2,3,2]~normal(0,1.5);

sigma~cauchy(0,3);

// likelihood for identified individuals

for (i in 1:nindi) {

if (lasti[i]>0) {

for (k in firsti[i]:lasti[i]) {

if(k>1) target+= (log(phi[i, k-1]));

if (yi[i,k] == 1) target+=(log(p[i,k]));

else target+=(log1m(p[i,k]));

}

}

target+=(log(chi[i,lasti[i]+1]));

}

// likelihood for non-identified individuals

for (i in 1:nindni) {

real log_like_male = 0;

real log_like_female = 0;

if (lastni[i]>0) {

for (k in firstni[i]:lastni[i]) {

if(k>1){

log_like_male += (log(phi_male[i, k-1]));

log_like_female += (log(phi_female[i, k-1]));

}

if (yni[i,k] == 1){

log_like_male+=(log(p_male[i,k]));

log_like_female+=(log(p_female[i,k]));

}

else{

log_like_male+=(log1m(p_male[i,k]));

log_like_female+=(log1m(p_female[i,k]));

}

}

}

log_like_male += (log(chi_male[i,lastni[i]+1]));

log_like_female += (log(chi_female[i,lastni[i]+1]));

target += log_mix(theta[i], log_like_male, log_like_female);

}

}

**Model 2b for reduced data set**

data {

int<lower=2> nocc; // number of capture events

int<lower=0> nind; // number of individuals with identified sex

int<lower=0,upper=2> y[nind,nocc]; // CH[i,k]: individual i captured at k

int<lower=0,upper=nocc-1> first[nind]; // year of first capture

int<lower=1, upper=2> sex[nind];

int<lower=1> year[nocc];

int<lower=1, upper=2> transients[nind, nocc]; // index of first capture occasion

}

transformed data {

int<lower=0,upper=nocc+1> last[nind]; // last[i]: ind i last capture

last = rep_array(0,nind);

for (i in 1:nind) {

for (k in first[i]:nocc) {

if (y[i,k] == 1) {

if (k > last[i]) last[i] = k;

}

}

}

}

parameters {

real<lower=0, upper=1> b0[2,nocc-1]; // intercept of p

real a0[2,2]; // intercept for phi

real<lower=0> sigma[2];

real yeareff[2, nocc-1];

}

transformed parameters {

real<lower=0,upper=1>p[nind,nocc]; // capture probability

real<lower=0,upper=1>phi[nind,nocc-1]; // survival probability

real<lower=0,upper=1>chi[nind,nocc+1]; // probability that an individual

// is never recaptured after its

// last capture

{

int k;

for(ii in 1:nind){

if (first[ii]>1) {

for (z in 1:(first[ii]-1)){

phi[ii,z] = 1;

}

}

for(tt in first[ii]:(nocc-1)) {

// linear predictor for phi:

phi[ii,tt] = inv_logit(a0[sex[ii], transients[ii,tt]] + sigma[sex[ii]]*yeareff[sex[ii],tt]);

}

}

for(i in 1:nind) {

// linear predictor for p for identified individuals

for(w in 1:first[i]){

p[i,w] = 1;

}

for(kk in (first[i]+1):nocc)

p[i,kk] = b0[sex[i],year[kk-1]];

chi[i,nocc+1] = 1.0;

k = nocc;

while (k > first[i]) {

chi[i,k] = (1 - phi[i,k-1]) + phi[i,k-1] * (1 - p[i,k]) * chi[i,k+1];

k = k - 1;

}

if (first[i]>1) {

for (u in 1:(first[i]-1)){

chi[i,u] = 0;

}

}

chi[i,first[i]] = (1 - p[i,first[i]]) * chi[i,first[i]+1];

}// close definition of transformed parameters for identified individuals

} // close block of transformed parameters exclusive parameter declarations

} // close transformed parameters

model {

// priors

for (g in 1:(nocc-1)){

b0[1,g]~beta(1,1);

b0[2,g]~beta(1,1);

yeareff[1, g]~normal(0,1);

yeareff[2, g]~normal(0,1);

}

a0[1,1]~normal(0,1.5);

a0[2,1]~normal(0,1.5);

a0[1,2]~normal(0,1.5);

a0[2,2]~normal(0,1.5);

sigma~cauchy(0,3);

// likelihood for identified individuals

for (i in 1:nind) {

if (last[i]>0) {

for (k in first[i]:last[i]) {

if(k>1) target+= (log(phi[i, k-1]));

if (y[i,k] == 1) target+=(log(p[i,k]));

else target+=(log1m(p[i,k]));

}

}

target+=(log(chi[i,last[i]+1]));

}

}

**Model 3a for full data set**

data {

int<lower=2> nocc; // number of capture events

int<lower=0> nindi; // number of individuals with identified sex

int<lower=0> nindni; // number of individuals with non-identified sex

int<lower=0,upper=2> yi[nindi,nocc]; // CH[i,k]: individual i captured at k

int<lower=0,upper=nocc-1> firsti[nindi]; // year of first capture

int<lower=0,upper=2> yni[nindni,nocc]; // CH[i,k]: individual i captured at k

int<lower=0,upper=nocc-1> firstni[nindni]; // year of first capture

int<lower=1, upper=2> sex[nindi];

int<lower=1, upper=3> juvi[nindi, nocc]; // 1 = ad, 2 = fully grown first year, 3 = pulli

int<lower=1, upper=3> juvni[nindni, nocc];

real tempsu[nocc-1];

real tempwi[nocc-1];

}

transformed data {

int<lower=0,upper=nocc+1> lasti[nindi]; // last capture

int<lower=0,upper=nocc+1> lastni[nindni]; // last capture

lasti = rep_array(0,nindi);

lastni = rep_array(0,nindni);

for (i in 1:nindi) {

for (k in firsti[i]:nocc) {

if (yi[i,k] == 1) {

if (k > lasti[i]) lasti[i] = k;

}

}

}

for (ii in 1:nindni) {

for (kk in firstni[ii]:nocc) {

if (yni[ii,kk] == 1) {

if (kk > lastni[ii]) lastni[ii] = kk;

}

}

}

}

parameters {

real<lower=0, upper=1> theta[nindni]; // probability of being male for non-identified individuals

real<lower=0, upper=1> b0[2,nocc-1]; // intercept of p

real a0[2,3]; // intercept for phi

real a1[2,3]; // intercept for phi

real a3[2,3]; // intercept for phi

}

transformed parameters {

real<lower=0,upper=1>p_male[nindni,nocc]; // capture probability

real<lower=0,upper=1>p_female[nindni,nocc]; // capture probability

real<lower=0,upper=1>p[nindi,nocc]; // capture probability

real<lower=0,upper=1>phi_male[nindni,nocc-1]; // survival probability

real<lower=0,upper=1>chi_male[nindni,nocc+1]; // probability that an individual

// is never recaptured after its

// last capture

real<lower=0,upper=1>phi_female[nindni,nocc-1]; // survival probability

real<lower=0,upper=1>chi_female[nindni,nocc+1]; // probability that an individual

// is never recaptured after its

// last capture

real<lower=0,upper=1>phi[nindi,nocc-1]; // survival probability

real<lower=0,upper=1>chi[nindi,nocc+1]; // probability that an individual

// is never recaptured after its

// last capture

{

int k;

int kk;

for(ii in 1:nindi){

if (firsti[ii]>1) {

for (z in 1:(firsti[ii]-1)){

phi[ii,z] = 1;

}

}

for(tt in firsti[ii]:(nocc-1)) {

// linear predictor for phi:

phi[ii,tt] = inv_logit(a0[sex[ii], juvi[ii,tt]] +

a1[sex[ii], juvi[ii,tt]] *tempsu[tt] +

a3[sex[ii], juvi[ii,tt]] *tempwi[tt]);

}

}

for(ii in 1:nindni){

if (firstni[ii]>1) {

for (z in 1:(firstni[ii]-1)){

phi_female[ii,z] = 1;

phi_male[ii,z] = 1;

}

}

for(tt in firstni[ii]:(nocc-1)) {

// linear predictor for phi:

phi_male[ii,tt] = inv_logit(a0[1, juvni[ii,tt]]+

a1[1, juvni[ii,tt]] *tempsu[tt] +

a3[1, juvni[ii,tt]] *tempwi[tt]);

phi_female[ii,tt] = inv_logit(a0[2, juvni[ii,tt]]+

a1[2, juvni[ii,tt]] *tempsu[tt] +

a3[2, juvni[ii,tt]] *tempwi[tt]);

}

}

for(i in 1:nindi) {

// linear predictor for p for identified individuals

for(w in 1:firsti[i]){

p[i,w] = 1;

}

for(kkk in (firsti[i]+1):nocc)

p[i,kkk] = b0[sex[i],kkk-1];

chi[i,nocc+1] = 1.0;

k = nocc;

while (k > firsti[i]) {

chi[i,k] = (1 - phi[i,k-1]) + phi[i,k-1] * (1 - p[i,k]) * chi[i,k+1];

k = k - 1;

}

if (firsti[i]>1) {

for (u in 1:(firsti[i]-1)){

chi[i,u] = 0;

}

}

chi[i,firsti[i]] = (1 - p[i,firsti[i]]) * chi[i,firsti[i]+1];

}// close definition of transformed parameters for identified individuals

for(i in 1:nindni) {

// linear predictor for p for non-identified individuals

for(w in 1:firstni[i]){

p_male[i,w] = 1;

p_female[i,w] = 1;

}

for(kkkk in (firstni[i]+1):nocc){

p_male[i,kkkk] = b0[1,kkkk-1];

p_female[i,kkkk] = b0[2,kkkk-1];

}

chi_male[i,nocc+1] = 1.0;

chi_female[i,nocc+1] = 1.0;

k = nocc;

while (k > firstni[i]) {

chi_male[i,k] = (1 - phi_male[i,k-1]) + phi_male[i,k-1] * (1 - p_male[i,k]) * chi_male[i,k+1];

chi_female[i,k] = (1 - phi_female[i,k-1]) + phi_female[i,k-1] * (1 - p_female[i,k]) * chi_female[i,k+1];

k = k - 1;

}

if (firstni[i]>1) {

for (u in 1:(firstni[i]-1)){

chi_male[i,u] = 0;

chi_female[i,u] = 0;

}

}

chi_male[i,firstni[i]] = (1 - p_male[i,firstni[i]]) * chi_male[i,firstni[i]+1];

chi_female[i,firstni[i]] = (1 - p_female[i,firstni[i]]) * chi_female[i,firstni[i]+1];

} // close definition of transformed parameters for non-identified individuals

} // close block of transformed parameters exclusive parameter declarations

} // close transformed parameters

model {

// priors

theta ~ beta(1, 1);

for (g in 1:(nocc-1)){

b0[1,g]~beta(1,1);

b0[2,g]~beta(1,1);

}

a0[1,1]~normal(0,1.5);

a0[1,2]~normal(0,1.5);

a0[2,1]~normal(0,1.5);

a0[2,2]~normal(0,1.5);

a0[1,3]~normal(0,1.5);

a0[2,3]~normal(0,1.5);

a1[1,1]~normal(0,3);

a1[1,2]~normal(0,3);

a1[2,1]~normal(0,3);

a1[2,2]~normal(0,3);

a1[1,3]~normal(0,3);

a1[2,3]~normal(0,3);

a3[1,1]~normal(0,3);

a3[1,2]~normal(0,3);

a3[2,1]~normal(0,3);

a3[2,2]~normal(0,3);

a3[1,3]~normal(0,3);

a3[2,3]~normal(0,3);

// likelihood for identified individuals

for (i in 1:nindi) {

if (lasti[i]>0) {

for (k in firsti[i]:lasti[i]) {

if(k>1) target+= (log(phi[i, k-1]));

if (yi[i,k] == 1) target+=(log(p[i,k]));

else target+=(log1m(p[i,k]));

}

}

target+=(log(chi[i,lasti[i]+1]));

}

// likelihood for non-identified individuals

for (i in 1:nindni) {

real log_like_male = 0;

real log_like_female = 0;

if (lastni[i]>0) {

for (k in firstni[i]:lastni[i]) {

if(k>1){

log_like_male += (log(phi_male[i, k-1]));

log_like_female += (log(phi_female[i, k-1]));

}

if (yni[i,k] == 1){

log_like_male+=(log(p_male[i,k]));

log_like_female+=(log(p_female[i,k]));

}

else{

log_like_male+=(log1m(p_male[i,k]));

log_like_female+=(log1m(p_female[i,k]));

}

}

}

log_like_male += (log(chi_male[i,lastni[i]+1]));

log_like_female += (log(chi_female[i,lastni[i]+1]));

target += log_mix(theta[i], log_like_male, log_like_female);

}

}

**Model 3a for reduced data set**

data {

int<lower=2> nocc; // number of capture events

int<lower=0> nind; // number of individuals with identified sex

int<lower=0,upper=2> y[nind,nocc]; // CH[i,k]: individual i captured at k

int<lower=0,upper=nocc-1> first[nind]; // year of first capture

int<lower=1, upper=2> sex[nind];

int<lower=1> year[nocc];

real tempsu[nocc-1]; // a covariate

real tempwi[nocc-1];

}

transformed data {

int<lower=0,upper=nocc+1> last[nind]; // last[i]: ind i last capture

last = rep_array(0,nind);

for (i in 1:nind) {

for (k in first[i]:nocc) {

if (y[i,k] == 1) {

if (k > last[i]) last[i] = k;

}

}

}

}

parameters {

real<lower=0, upper=1> b0[2,nocc-1]; // intercept of p

real a0[2]; // intercept for phi

real a1[2]; // coefficient for phi

real a2[2]; // coefficient for phi

}

transformed parameters {

real<lower=0,upper=1>p[nind,nocc]; // capture probability

real<lower=0,upper=1>phi[nind,nocc-1]; // survival probability

real<lower=0,upper=1>chi[nind,nocc+1]; // probability that an individual

// is never recaptured after its

// last capture

{

int k;

for(ii in 1:nind){

if (first[ii]>1) {

for (z in 1:(first[ii]-1)){

phi[ii,z] = 1;

}

}

for(tt in first[ii]:(nocc-1)) {

// linear predictor for phi:

phi[ii,tt] = inv_logit(a0[sex[ii]]+ a1[sex[ii]]*tempsu[tt]+a2[sex[ii]]*tempwi[tt]);

}

}

for(i in 1:nind) {

// linear predictor for p for identified individuals

for(w in 1:first[i]){

p[i,w] = 1;

}

for(kk in (first[i]+1):nocc)

p[i,kk] = b0[sex[i],year[kk-1]];

chi[i,nocc+1] = 1.0;

k = nocc;

while (k > first[i]) {

chi[i,k] = (1 - phi[i,k-1]) + phi[i,k-1] * (1 - p[i,k]) * chi[i,k+1];

k = k - 1;

}

if (first[i]>1) {

for (u in 1:(first[i]-1)){

chi[i,u] = 0;

}

}

chi[i,first[i]] = (1 - p[i,first[i]]) * chi[i,first[i]+1];

}// close definition of transformed parameters for identified individuals

} // close block of transformed parameters exclusive parameter declarations

} // close transformed parameters

model {

// priors

for (g in 1:(nocc-1)){

b0[1,g]~beta(1,1);

b0[2,g]~beta(1,1);

}

a0[1]~normal(0,1.5);

a1[1]~normal(0,3);

a2[1]~normal(0,3);

a0[2]~normal(0,1.5);

a1[2]~normal(0,3);

a2[2]~normal(0,3);

// likelihood for identified individuals

for (i in 1:nind) {

if (last[i]>0) {

for (k in first[i]:last[i]) {

if(k>1) target+= (log(phi[i, k-1]));

if (y[i,k] == 1) target+=(log(p[i,k]));

else target+=(log1m(p[i,k]));

}

}

target+=(log(chi[i,last[i]+1]));

}

}

**Model 4 for full data set**

data {

int<lower=2> nocc; // number of capture events

int<lower=0> nindi; // number of individuals with identified sex

int<lower=0> nindni; // number of individuals with non-identified sex

int<lower=0,upper=2> yi[nindi,nocc]; // CH[i,k]: individual i captured at k

int<lower=0,upper=nocc-1> firsti[nindi]; // year of first capture

int<lower=0,upper=2> yni[nindni,nocc]; // CH[i,k]: individual i captured at k

int<lower=0,upper=nocc-1> firstni[nindni]; // year of first capture

int<lower=1, upper=2> sex[nindi];

int<lower=1, upper=3> juvi[nindi, nocc]; // 1 = ad, 2 = fully grown first year, 3 = pulli

int<lower=1, upper=3> juvni[nindni, nocc];

real tempsu[nocc-1];

real tempwi[nocc-1];

real rainsu[nocc-1];

real rainwi[nocc-1];

}

transformed data {

int<lower=0,upper=nocc+1> lasti[nindi]; // last[i]: ind i last capture

int<lower=0,upper=nocc+1> lastni[nindni]; // last[i]: ind i last capture

lasti = rep_array(0,nindi);

lastni = rep_array(0,nindni);

for (i in 1:nindi) {

for (k in firsti[i]:nocc) {

if (yi[i,k] == 1) {

if (k > lasti[i]) lasti[i] = k;

}

}

}

for (ii in 1:nindni) {

for (kk in firstni[ii]:nocc) {

if (yni[ii,kk] == 1) {

if (kk > lastni[ii]) lastni[ii] = kk;

}

}

}

}

parameters {

real<lower=0, upper=1> theta[nindni]; // probability of being male for non-identified individuals

real<lower=0, upper=1> b0[2,nocc-1]; // intercept of p

real a0[2,3]; // intercept for phi

real a1[2,3]; //

real a2[2,3]; //

real a3[2,3]; //

real a4[2,3]; //

}

transformed parameters {

real<lower=0,upper=1>p_male[nindni,nocc]; // capture probability

real<lower=0,upper=1>p_female[nindni,nocc]; // capture probability

real<lower=0,upper=1>p[nindi,nocc]; // capture probability

real<lower=0,upper=1>phi_male[nindni,nocc-1]; // survival probability

real<lower=0,upper=1>chi_male[nindni,nocc+1]; // probability that an individual

// is never recaptured after its

// last capture

real<lower=0,upper=1>phi_female[nindni,nocc-1]; // survival probability

real<lower=0,upper=1>chi_female[nindni,nocc+1]; // probability that an individual

// is never recaptured after its

// last capture

real<lower=0,upper=1>phi[nindi,nocc-1]; // survival probability

real<lower=0,upper=1>chi[nindi,nocc+1]; // probability that an individual

// is never recaptured after its

// last capture

{

int k;

int kk;

for(ii in 1:nindi){

if (firsti[ii]>1) {

for (z in 1:(firsti[ii]-1)){

phi[ii,z] = 1;

}

}

for(tt in firsti[ii]:(nocc-1)) {

// linear predictor for phi:

phi[ii,tt] = inv_logit(a0[sex[ii], juvi[ii,tt]] +

a1[sex[ii], juvi[ii,tt]] *tempsu[tt] +

a2[sex[ii], juvi[ii,tt]] *rainsu[tt] +

a3[sex[ii], juvi[ii,tt]] *tempwi[tt] +

a4[sex[ii], juvi[ii,tt]] *rainwi[tt]);

}

}

for(ii in 1:nindni){

if (firstni[ii]>1) {

for (z in 1:(firstni[ii]-1)){

phi_female[ii,z] = 1;

phi_male[ii,z] = 1;

}

}

for(tt in firstni[ii]:(nocc-1)) {

// linear predictor for phi:

phi_male[ii,tt] = inv_logit(a0[1, juvni[ii,tt]]+

a1[1, juvni[ii,tt]] *tempsu[tt] +

a2[1, juvni[ii,tt]] *rainsu[tt] +

a3[1, juvni[ii,tt]] *tempwi[tt] +

a4[1, juvni[ii,tt]] *rainwi[tt]);

phi_female[ii,tt] = inv_logit(a0[2, juvni[ii,tt]]+

a1[2, juvni[ii,tt]] *tempsu[tt] +

a2[2, juvni[ii,tt]] *rainsu[tt] +

a3[2, juvni[ii,tt]] *tempwi[tt] +

a4[2, juvni[ii,tt]] *rainwi[tt]);

}

}

for(i in 1:nindi) {

// linear predictor for p for identified individuals

for(w in 1:firsti[i]){

p[i,w] = 1;

}

for(kkk in (firsti[i]+1):nocc)

p[i,kkk] = b0[sex[i],kkk-1];

chi[i,nocc+1] = 1.0;

k = nocc;

while (k > firsti[i]) {

chi[i,k] = (1 - phi[i,k-1]) + phi[i,k-1] * (1 - p[i,k]) * chi[i,k+1];

k = k - 1;

}

if (firsti[i]>1) {

for (u in 1:(firsti[i]-1)){

chi[i,u] = 0;

}

}

chi[i,firsti[i]] = (1 - p[i,firsti[i]]) * chi[i,firsti[i]+1];

}// close definition of transformed parameters for identified individuals

for(i in 1:nindni) {

// linear predictor for p for non-identified individuals

for(w in 1:firstni[i]){

p_male[i,w] = 1;

p_female[i,w] = 1;

}

for(kkkk in (firstni[i]+1):nocc){

p_male[i,kkkk] = b0[1,kkkk-1];

p_female[i,kkkk] = b0[2,kkkk-1];

}

chi_male[i,nocc+1] = 1.0;

chi_female[i,nocc+1] = 1.0;

k = nocc;

while (k > firstni[i]) {

chi_male[i,k] = (1 - phi_male[i,k-1]) + phi_male[i,k-1] * (1 - p_male[i,k]) * chi_male[i,k+1];

chi_female[i,k] = (1 - phi_female[i,k-1]) + phi_female[i,k-1] * (1 - p_female[i,k]) * chi_female[i,k+1];

k = k - 1;

}

if (firstni[i]>1) {

for (u in 1:(firstni[i]-1)){

chi_male[i,u] = 0;

chi_female[i,u] = 0;

}

}

chi_male[i,firstni[i]] = (1 - p_male[i,firstni[i]]) * chi_male[i,firstni[i]+1];

chi_female[i,firstni[i]] = (1 - p_female[i,firstni[i]]) * chi_female[i,firstni[i]+1];

} // close definition of transformed parameters for non-identified individuals

} // close block of transformed parameters exclusive parameter declarations

} // close transformed parameters

model {

// priors

theta ~ beta(1, 1);

for (g in 1:(nocc-1)){

b0[1,g]~beta(1,1);

b0[2,g]~beta(1,1);

}

a0[1,1]~normal(0,1.5);

a0[1,2]~normal(0,1.5);

a0[2,1]~normal(0,1.5);

a0[2,2]~normal(0,1.5);

a0[1,3]~normal(0,1.5);

a0[2,3]~normal(0,1.5);

a1[1,1]~normal(0,3);

a1[1,2]~normal(0,3);

a1[2,1]~normal(0,3);

a1[2,2]~normal(0,3);

a1[1,3]~normal(0,3);

a1[2,3]~normal(0,3);

a2[1,1]~normal(0,3);

a2[1,2]~normal(0,3);

a2[2,1]~normal(0,3);

a2[2,2]~normal(0,3);

a2[1,3]~normal(0,3);

a2[2,3]~normal(0,3);

a3[1,1]~normal(0,3);

a3[1,2]~normal(0,3);

a3[2,1]~normal(0,3);

a3[2,2]~normal(0,3);

a3[1,3]~normal(0,3);

a3[2,3]~normal(0,3);

a4[1,1]~normal(0,3);

a4[1,2]~normal(0,3);

a4[2,1]~normal(0,3);

a4[2,2]~normal(0,3);

a4[1,3]~normal(0,3);

a4[2,3]~normal(0,3);

// likelihood for identified individuals

for (i in 1:nindi) {

if (lasti[i]>0) {

for (k in firsti[i]:lasti[i]) {

if(k>1) target+= (log(phi[i, k-1]));

if (yi[i,k] == 1) target+=(log(p[i,k]));

else target+=(log1m(p[i,k]));

}

}

target+=(log(chi[i,lasti[i]+1]));

}

// likelihood for non-identified individuals

for (i in 1:nindni) {

real log_like_male = 0;

real log_like_female = 0;

if (lastni[i]>0) {

for (k in firstni[i]:lastni[i]) {

if(k>1){

log_like_male += (log(phi_male[i, k-1]));

log_like_female += (log(phi_female[i, k-1]));

}

if (yni[i,k] == 1){

log_like_male+=(log(p_male[i,k]));

log_like_female+=(log(p_female[i,k]));

}

else{

log_like_male+=(log1m(p_male[i,k]));

log_like_female+=(log1m(p_female[i,k]));

}

}

}

log_like_male += (log(chi_male[i,lastni[i]+1]));

log_like_female += (log(chi_female[i,lastni[i]+1]));

target += log_mix(theta[i], log_like_male, log_like_female);

}

}

**Model 4 for reduced data set**

data {

int<lower=2> nocc; // number of capture events

int<lower=0> nind; // number of individuals with identified sex

int<lower=0,upper=2> y[nind,nocc]; // CH[i,k]: individual i captured at k

int<lower=0,upper=nocc-1> first[nind]; // year of first capture

int<lower=1, upper=2> sex[nind];

int<lower=1> year[nocc];

real tempsu[nocc-1]; // a covariate

real tempwi[nocc-1];

real rainsu[nocc-1];

real rainwi[nocc-1];

}

transformed data {

int<lower=0,upper=nocc+1> last[nind]; // last[i]: ind i last capture

last = rep_array(0,nind);

for (i in 1:nind) {

for (k in first[i]:nocc) {

if (y[i,k] == 1) {

if (k > last[i]) last[i] = k;

}

}

}

}

parameters {

real<lower=0, upper=1> b0[2,nocc-1]; // intercept of p

real a0[2]; // intercept for phi

real a1[2]; // coefficient for phi

real a2[2]; // coefficient for phi

real a3[2]; // coefficient for phi

real a4[2]; // coefficient for phi

}

transformed parameters {

real<lower=0,upper=1>p[nind,nocc]; // capture probability

real<lower=0,upper=1>phi[nind,nocc-1]; // survival probability

real<lower=0,upper=1>chi[nind,nocc+1]; // probability that an individual

// is never recaptured after its

// last capture

{

int k;

for(ii in 1:nind){

if (first[ii]>1) {

for (z in 1:(first[ii]-1)){

phi[ii,z] = 1;

}

}

for(tt in first[ii]:(nocc-1)) {

// linear predictor for phi:

phi[ii,tt] = inv_logit(a0[sex[ii]]+ a1[sex[ii]]*tempsu[tt]+

a2[sex[ii]]*tempwi[tt]+a3[sex[ii]]*rainsu[tt] +

a4[sex[ii]]*rainwi[tt]); //

}

}

for(i in 1:nind) {

// linear predictor for p for identified individuals

for(w in 1:first[i]){

p[i,w] = 1;

}

for(kk in (first[i]+1):nocc)

p[i,kk] = b0[sex[i],year[kk-1]];

chi[i,nocc+1] = 1.0;

k = nocc;

while (k > first[i]) {

chi[i,k] = (1 - phi[i,k-1]) + phi[i,k-1] * (1 - p[i,k]) * chi[i,k+1];

k = k - 1;

}

if (first[i]>1) {

for (u in 1:(first[i]-1)){

chi[i,u] = 0;

}

}

chi[i,first[i]] = (1 - p[i,first[i]]) * chi[i,first[i]+1];

}// close definition of transformed parameters for identified individuals

} // close block of transformed parameters exclusive parameter declarations

} // close transformed parameters

model {

// priors

for (g in 1:(nocc-1)){

b0[1,g]~beta(1,1);

b0[2,g]~beta(1,1);

}

a0[1]~normal(0,1.5);

a0[2]~normal(0,1.5);

a1[1]~normal(0,3);

a1[2]~normal(0,3);

a2[1]~normal(0,3);

a2[2]~normal(0,3);

a3[1]~normal(0,3);

a3[2]~normal(0,3);

a4[1]~normal(0,3);

a4[2]~normal(0,3);

// likelihood for identified individuals

for (i in 1:nind) {

if (last[i]>0) {

for (k in first[i]:last[i]) {

if(k>1) target+= (log(phi[i, k-1]));

if (y[i,k] == 1) target+=(log(p[i,k]));

else target+=(log1m(p[i,k]));

}

}

target+=(log(chi[i,last[i]+1]));

}

}

**Model 5 for full data set**

data {

int<lower=2> nocc; // number of capture events

int<lower=0> nindi; // number of individuals with identified sex

int<lower=0> nindni; // number of individuals with non identified sex

int<lower=0,upper=1> yi[nindi,nocc]; // CH[i,k]: individual i captured at k

int<lower=0,upper=1> yni[nindni,nocc]; // CH[i,k]: individual i captured at k

int<lower=0,upper=nocc-1> firsti[nindi]; // occasion of first capture

int<lower=0,upper=nocc-1> firstni[nindni]; // occasion of first capture

int<lower=1,upper=3> season[nocc]; // season of capture occasion

int<lower=1, upper=2> sex[nindi];

int<lower=0, upper=1> juvi[nindi,nocc];

int<lower=0, upper=1> juvni[nindni,nocc];

int<lower=0> year[nocc];

int<lower=0> nyears;

}

transformed data {

int<lower=0,upper=nocc+1> lasti[nindi]; // last[i]: ind i last capture

int<lower=0,upper=nocc+1> lastni[nindni]; // last[i]: ind i last capture

lasti = rep_array(0,nindi);

for (i in 1:nindi) {

for (k in firsti[i]:nocc) {

if (yi[i,k] == 1) {

if (k > lasti[i]) lasti[i] = k;

}

}

}

lastni = rep_array(0,nindni);

for (ii in 1:nindni) {

for (kk in firstni[ii]:nocc) {

if (yni[ii,kk] == 1) {

if (kk > lastni[ii]) lastni[ii] = kk;

}

}

}

}

parameters {

real<lower=0, upper=1> theta[nindni]; // probability of being male for non-identified individuals

real b0[3,3]; // intercept of p

real<lower=0, upper=1> a[3,3]; // coefficients for phi

real<lower=0> sigmayp;

real yeareffp[nyears];

}

transformed parameters {

real<lower=0,upper=1>p[nindi,nocc]; // capture probability

real<lower=0,upper=1>phi[nindi,nocc-1]; // survival probability

real<lower=0,upper=1>chi[nindi,nocc+1]; // probability that an individual

// is never recaptured after its

// last capture

real<lower=0,upper=1>p_male[nindni,nocc]; // capture probability

real<lower=0,upper=1>p_female[nindni,nocc]; // capture probability

real<lower=0,upper=1>phi_male[nindni,nocc-1]; // survival probability

real<lower=0,upper=1>phi_female[nindni,nocc-1]; // survival probability

real<lower=0,upper=1>chi_male[nindni,nocc+1]; //

real<lower=0,upper=1>chi_female[nindni,nocc+1]; //

{

// for identified individuals

int k;

for(ii in 1:nindi){

if (firsti[ii]>1) {

for (z in 1:(firsti[ii]-1)){

phi[ii,z] = 1;

}

}

for(tt in firsti[ii]:(nocc-1)) {

// linear predictor for phi:

if(juvi[ii, tt] ==1){

phi[ii,tt] = a[1,season[tt]];

}

if(juvi[ii, tt] ==0){

phi[ii,tt] = a[sex[ii]+1,season[tt]]; //inv_logit(...+ ryp[year[tt]]*sigmapy)

}

}

}

for(i in 1:nindi) {

// linear predictor for p

for(w in 1:firsti[i]){

p[i,w] = 1;

}

for(kk in (firsti[i]+1):nocc){

if(juvi[i,kk]==1) {

p[i,kk] = inv_logit(b0[1,season[kk]] + sigmayp*yeareffp[year[kk]]);

}

if(juvi[i,kk]==0) {

p[i,kk] = inv_logit(b0[sex[i]+1,season[kk]]+ sigmayp*yeareffp[year[kk]]);

}

}

chi[i,nocc+1] = 1.0;

k = nocc;

while (k > firsti[i]) {

chi[i,k] = (1 - phi[i,k-1]) + phi[i,k-1] * (1 - p[i,k]) * chi[i,k+1];

k = k - 1;

}

if (firsti[i]>1) {

for (u in 1:(firsti[i]-1)){

chi[i,u] = 0;

}

}

chi[i,firsti[i]] = (1 - p[i,firsti[i]]) * chi[i,firsti[i]+1];

}

}

// for non-identified sex

for(ii in 1:nindni){

if (firstni[ii]>1) {

for (z in 1:(firstni[ii]-1)){

phi_male[ii,z] = 1;

phi_female[ii,z] = 1;

}

}

for(tt in firstni[ii]:(nocc-1)) {

// linear predictor for phi:

if(juvni[ii, tt] ==1){

phi_male[ii,tt] = a[1,season[tt]]; //inv_logit(...+ ryp[year[tt]]*sigmapy)

phi_female[ii,tt] = a[1,season[tt]]; //inv_logit(...+ ryp[year[tt]]*sigmapy)

}

if(juvni[ii, tt] ==0){

phi_male[ii,tt] = a[2,season[tt]]; //inv_logit(...+ ryp[year[tt]]*sigmapy)

phi_female[ii,tt] = a[3,season[tt]]; //inv_logit(...+ ryp[year[tt]]*sigmapy)

}

}

}

for(i in 1:nindni) {

// linear predictor for p

for(w in 1:firstni[i]){

p_male[i,w] = 1;

p_female[i,w] = 1;

}

for(kk in (firstni[i]+1):nocc){

if(juvni[i,kk]==1) {

p_male[i,kk] = inv_logit(b0[1,season[kk]]+ sigmayp*yeareffp[year[kk]]);

p_female[i,kk] = inv_logit(b0[1,season[kk]]+ sigmayp*yeareffp[year[kk]]);

}

if(juvni[i,kk]==0) {

p_male[i,kk] = inv_logit(b0[2,season[kk]]+ sigmayp*yeareffp[year[kk]]);

p_female[i,kk] = inv_logit(b0[3,season[kk]]+ sigmayp*yeareffp[year[kk]]);

}

}

chi_male[i,nocc+1] = 1.0;

chi_female[i,nocc+1] = 1.0;

{ // for the q-loop

int q=nocc;

while (q > firstni[i]) {

chi_male[i,q] = (1 - phi_male[i,q-1]) + phi_male[i,q-1] * (1 - p_male[i,q]) * chi_male[i,q+1];

chi_female[i,q] = (1 - phi_female[i,q-1]) + phi_female[i,q-1] * (1 - p_female[i,q]) * chi_female[i,q+1];

q = q - 1;

}

}// close the q-loop

if (firstni[i]>1) {

for (u in 1:(firstni[i]-1)){

chi_male[i,u] = 0;

chi_female[i,u] = 0;

}

}

chi_male[i,firstni[i]] = (1 - p_male[i,firstni[i]]) * chi_male[i,firstni[i]+1];

chi_female[i,firstni[i]] = (1 - p_female[i,firstni[i]]) * chi_female[i,firstni[i]+1];

}

}

model {

// priors

theta ~ beta(1, 1);

for (g in 1:3){

b0[1,g] ~ normal(0,1.5);

b0[2,g] ~ normal(0,1.5);

b0[3,g] ~ normal(0,1.5);

}

// priors

a[1,1]~beta(3.6, 1.2);

a[1,2]~beta(3.6, 1.2);

a[1,3]~beta(3.6, 1.2);

a[2,1]~beta(3.6, 1.2);

a[2,2]~beta(3.6, 1.2);

a[2,3]~beta(3.6, 1.2);

a[3,1]~beta(3.6, 1.2);

a[3,2]~beta(3.6, 1.2);

a[3,3]~beta(3.6, 1.2);

// random effects

sigmayp~cauchy(0,3);

for(h in 1:nyears){

yeareffp[h] ~ normal(0,1);

}

// likelihood for identified individuals

for (i in 1:nindi) {

if (lasti[i]>0) {

for (k in firsti[i]:lasti[i]) {

if(k>1) target+= (log(phi[i, k-1]));

if (yi[i,k] == 1) target+=(log(p[i,k]));

else target+=(log1m(p[i,k]));

}

}

target+=(log(chi[i,lasti[i]+1]));

}

// likelihood for non-identified individuals

for (i in 1:nindni) {

real log_like_male = 0;

real log_like_female = 0;

if (lastni[i]>0) {

for (k in firstni[i]:lastni[i]) {

if(k>1){

log_like_male += (log(phi_male[i, k-1]));

log_like_female += (log(phi_female[i, k-1]));

}

if (yni[i,k] == 1){

log_like_male +=(log(p_male[i,k]));

log_like_female +=(log(p_female[i,k]));

}

else {

log_like_male +=(log1m(p_male[i,k]));

log_like_female +=(log1m(p_female[i,k]));

}

}

}

log_like_male += (log(chi_male[i,lastni[i]+1]));

log_like_female += (log(chi_female[i,lastni[i]+1]));

target += log_mix(theta[i], log_like_male, log_like_female);

}

}

**Model 5 for reduced data set**

data {

int<lower=2> nocc; // number of capture events

int<lower=0> nind; // number of individuals with identified sex

int<lower=0,upper=1> y[nind,nocc]; // CH[i,k]: individual i captured at k

int<lower=0,upper=nocc-1> first[nind]; // occasion of first capture

int<lower=1,upper=3> season[nocc]; // season of capture occasion

int<lower=1, upper=2> sex[nind];

int<lower=0> year[nocc];

int<lower=0> nyears;

}

transformed data {

int<lower=0,upper=nocc+1> last[nind]; // last[i]: ind i last capture

last = rep_array(0,nind);

for (i in 1:nind) {

for (k in first[i]:nocc) {

if (y[i,k] == 1) {

if (k > last[i]) last[i] = k;

}

}

}

}

parameters {

real b0[2,3]; // intercept of p

real<lower=0, upper=1> a[2,3]; // coefficients for phi

real<lower=0> sigmayp;

real yeareffp[nyears];

}

transformed parameters {

real<lower=0,upper=1>p[nind,nocc]; // capture probability

real<lower=0,upper=1>phi[nind,nocc-1]; // survival probability

real<lower=0,upper=1>chi[nind,nocc+1]; // probability that an individual

// is never recaptured after its

// last capture

{

int k;

for(ii in 1:nind){

if (first[ii]>1) {

for (z in 1:(first[ii]-1)){

phi[ii,z] = 1;

}

}

for(tt in first[ii]:(nocc-1)) {

// linear predictor for phi:

phi[ii,tt] = a[sex[ii],season[tt]]; //inv_logit(...+ ryp[year[tt]]*sigmapy)

}

}

for(i in 1:nind) {

// linear predictor for p

for(w in 1:first[i]){

p[i,w] = 1;

}

for(kk in (first[i]+1):nocc){

p[i,kk] = inv_logit(b0[sex[i],season[kk]]+ sigmayp*yeareffp[year[kk]]);

}

chi[i,nocc+1] = 1.0;

k = nocc;

while (k > first[i]) {

chi[i,k] = (1 - phi[i,k-1]) + phi[i,k-1] * (1 - p[i,k]) * chi[i,k+1];

k = k - 1;

}

if (first[i]>1) {

for (u in 1:(first[i]-1)){

chi[i,u] = 0;

}

}

chi[i,first[i]] = (1 - p[i,first[i]]) * chi[i,first[i]+1];

}

}

}

model {

// priors

for (g in 1:3){

b0[1,g] ~ normal(0,1.5);

b0[2,g] ~ normal(0,1.5);

}

// priors

a[1,1]~beta(3.6, 1.2);

a[1,2]~beta(3.6, 1.2);

a[1,3]~beta(3.6, 1.2);

a[2,1]~beta(3.6, 1.2);

a[2,2]~beta(3.6, 1.2);

a[2,3]~beta(3.6, 1.2);

// random effects

sigmayp~cauchy(0,3);

for(h in 1:nyears){

yeareffp[h] ~ normal(0,1);

}

for (i in 1:nind) {

if (last[i]>0) {

for (k in first[i]:last[i]) {

if(k>1) target+= (log(phi[i, k-1]));

if (y[i,k] == 1) target+=(log(p[i,k]));

else target+=(log1m(p[i,k]));

}

}

target+=(log(chi[i,last[i]+1]));

}

}
